# Semantic Similarity‐Informed Bayesian Borrowing for Quantitative Signal Detection of Adverse Events

**DOI:** 10.1002/sim.70757

**Published:** 2026-10-15

**Authors:** François Haguinet, Jeffery L. Painter, Gregory E. Powell, Andrea Callegaro, Andrew Bate

**Affiliations:** ^1^ GSK Wavre Belgium; ^2^ GSK Durham North Carolina USA; ^3^ GSK London UK; ^4^ London School of Hygiene and Tropical Medicine London UK

**Keywords:** Bayesian borrowing, drug safety, pharmacovigilance, semantic similarity, signal detection

## Abstract

We present a Bayesian dynamic borrowing approach to enhance quantitative identification of adverse events in spontaneous reporting systems. The method embeds a robust meta‐analytic predictive prior and incorporates semantic similarity measures (SSMs) to enable weighted information sharing from clinically similar MedDRA preferred terms (PTs) to the target PT. This continuous similarity‐based borrowing overcomes limitations of rigid hierarchical grouping in disproportionality analysis (DPA). Using data from the FDA Adverse Event Reporting System and a time‐stamped reference set, we evaluate our approach—termed IC SSM—against the current standard approach of “traditional” information component (IC) analysis and borrowing at the MedDRA HLGT level (IC HLGT). The primary IC SSM approach at the standard threshold (2.5% posterior quantile >0) detected true signals over five months earlier than traditional IC. It showed higher sensitivity (57.0%) but lower precision (14.5%) than traditional IC (50.1%, 16.1%), while outperforming IC HLGT (55.7%, 13.9%). In subsequent analyses, even when tightening the alerting quantile of IC SSM to 1% to improve precision (16.4%), superior sensitivity (51.4%) was retained compared to traditional IC. Similarly, relaxing traditional IC's quantile to 5% yielded only 55.6% sensitivity with 14.2% precision, failing to match IC SSM's performance or timeliness and confirming its inherent advantage. IC SSM's superior timeliness and sensitivity stem from integrating clinically meaningful information, making it a scalable and context‐aware enhancement to traditional DPA. The method has potential for exploratory analysis in sparse datasets, and refinements to the borrowing strategies and similarity metrics could yield further performance improvements.

## 1 Introduction

Post‐marketing safety surveillance of medicines and vaccines during routine healthcare delivery is essential for identifying emerging safety concerns and evaluating risks previously identified or hypothesized during development [1]. This process ensures that marketed products continue to reflect an accurate benefit‐risk profile. Spontaneous reporting of individual case safety reports (ICSRs) has long served as the primary data source for detecting new safety signals [2]. While clinical review remains fundamental, the increasing volume and complexity of safety data have elevated the role of quantitative methods in pharmacovigilance (PV).

Quantitative analysis of ICSRs was first proposed in the late 1960s and 1970s [3, 4]. Although, early applications were sporadic and often limited to specific product issues [5, 6], the late 1990s marked a turning point, with routine use of these methods as a guide to where to focus clinical review, driven by increasing data availability and advances in computation [7]. While some quantitative signal detection approaches incorporate external data such as sales or prescription volumes to construct background rates [8, 9], the most widely used methods rely solely on ICSR data due to their practicality and superior performance.^1^ [10, 11, 12, 13, 14, 15, 16]. The standard methods and their implementations—introduced in the late 1990s and early 2000s—including the information component (IC) [17]—are based on point estimates estimated from two‐by‐two contingency tables with 95% confidence limits and, despite their simplicity, remain the foundation of quantitative signal detection [17, 18, 19, 20], despite their simplicity and often perform quite similarly [20, 21]. Notably, a multi‐database comparison from the IMI PROTECT project showed that variation in signal detection performance across the standard methods was more influenced by the chosen detection threshold than by the specific algorithm employed [22, 23]. More complex techniques have since been proposed, they have not consistently outperformed these foundational methods in real‐world settings [22].

Nevertheless, disproportionality analysis (DPA) using these scores has notable limitations. The underlying data are noisy and subject to non‐random missingness, and the independent treatment of adverse event (AE) terms limits their ability to capture clinically similar cases that may share an underlying mechanism [24, 25]. Addressing this limitation requires a more effective way to cluster related AEs for signal detection.

The medical dictionary for regulatory activities (MedDRA) [26] is an international hierarchical terminology for coding AEs in clinical trials and post‐marketing ICSRs. It facilitates consistency in AE classification and enables grouping of related events via its tree structure, which includes levels of specificity from preferred terms (PTs) to higher‐level groupings. While useful, MedDRA's rigid hierarchy often fails to capture nuanced clinical relationships between terms [27]. For instance, the PT *Eyelid oedema* and the PT *Periorbital oedema* are located far away from each other in the hierarchy. Another example is the PT *Pneumonitis NOS* and the PT *Pneumonia NOS*.

Standard DPA methods treat MedDRA PTs independently, unless they are grouped a priori under the MedDRA hierarchy, where all included PTs are effectively treated as synonymous. This approach can lead to missed signals when related but distinct PTs are analyzed in isolation or to diluted signals when overly broad terms group unrelated AEs. Although, general groupings like high‐level group terms (HLGTs) or standardized MedDRA queries (SMQs) are sometimes used during post hoc clinical review, there is little evidence that higher‐level aggregation improves algorithmic performance of signal detection [28]. As a result, most automated analyses rely on PT or lower level term (LLT) granularity, both of which exist at the same hierarchical level within MedDRA [29].

To address these difficulties in outcome definition granularity and the associated challenge of capturing the nuanced continuum of clinical relatedness, alternative methodologies have emerged. For example, TreeScan has been used to test all possible AE terms across every level of the predefined MedDRA hierarchy while accounting for multiple comparisons, but it remains constrained to this rigid hierarchy and does not incorporate semantic similarity [30]. Similarly, approaches such as the regression‐adjusted gamma poisson shrinker (RGPS) method [31] and large‐scale regression‐based pattern discovery techniques [32], including regression models that explicitly capture co‐reporting interdependencies among similar events, aim to uncover patterns across related outcomes that may be missed when AEs are evaluated in isolation. Bayesian borrowing, especially when informed by semantic similarity, offers a potential solution to the same limitation [33]. Previous applications to ICSRs, particularly for vaccines, have demonstrated benefits when borrowing information across PTs within the same system organ class (SOC) [34]. However, these methods often rely on binary definitions of similarity: PTs are either allowed to borrow from each other or not. This binary framing fails to reflect the continuum of clinical relatedness among AEs. In reality, AEs vary in their informativeness for one another, and a continuous weighting scheme would allow more nuanced borrowing.

Semantic similarity provides a mechanism for introducing such gradation, enabling context‐sensitive borrowing across PTs. Although, its application in PV remains limited, some related work has explored data‐driven clustering of terms that frequently co‐occur [35, 36, 37]. Nonetheless, we deliberately opted not to use these methods in our study. By relying solely on combinations of established ontologies, our semantic similarity measure (SSM) is independent of the choice of data source, avoiding the limitations of co‐reporting patterns and enabling consistent weights to apply across different data sets aiding transparency.

Bayesian dynamic borrowing (BDB) has gained traction in clinical trials for integrating historical control data based on similarity to current study data. Pocock [38] pioneered this approach, which has since evolved into several frameworks, including the power prior [39], modified power prior [40, 41], the meta‐analytic predictive (MAP) method [42], and the commensurate prior [43]. Schmidli et al. [44] enhanced the MAP method by introducing a robust MAP prior, incorporating a vague component to mitigate the impact of discordant historical data.

This manuscript presents, to our knowledge for the first time, a novel adaptation of BDB that incorporates semantic similarity for spontaneous reporting systems (SRSs), using these similarity scores to guide information sharing across MedDRA PTs. Throughout this work, we refer to this method as IC SSM—a semantically informed extension of the IC analysis framework. IC SSM embeds a robust MAP prior within a Bayesian hierarchical model, allowing for dynamically weighted borrowing from PTs that are clinically similar to the PT of interest. This continuous, data‐driven borrowing approach replaces rigid hierarchical structures with context‐sensitive inference and aims to preserve statistical power while reducing noise from clinically irrelevant terms. We evaluate the performance of IC SSM in comparison to both traditional IC analysis and IC analysis using MedDRA HLGT borrowing.

Using data from the FDA adverse event reporting system (FAERS) from 2015 to 2019, we conduct evaluations against a reference set derived from FDA product label updates to assess the method's ability to detect signals prior to official labeling. Sensitivity analyses are also conducted to explore the impact of key model parameters. This work contributes to the advancement of PV methodologies by addressing a long‐standing challenge in AE aggregation, aiming to improve the accuracy and timeliness of early signal detection in post‐marketing surveillance.

## 2 Methods

### 2.1 Data

This study utilized data from FAERS, specifically from the third quarter 2022 release (2022Q3). To mitigate potential confounding effects introduced by the COVID‐19 pandemic, all reports submitted after December 31, 2019, were excluded. This time window (from 2015 to 2019) was selected to align with complementary data sources, such as the Sentinel Initiative [45, 46], allowing for future comparative analyses using a consistent dataset.

Our analysis focused on a curated cohort of 69 drugs approved by the FDA between January 1, 2015, and December 31, 2016, under submission Type 1 (new molecular entity). These drugs were identified using our PVLens platform, which processes and integrates structured product labeling (SPL) documents to generate harmonized substance‐level identifiers [47]. PVLens achieves this by mapping SPL‐derived information—such as RxNorm codes and national drug codes (NDCs)—into standardized representations, further aligned via the UMLS MTHSPL (FDA structured product labeling source information) table to ensure consistent tracking across label versions [48].

To link FAERS reports to these substances, we developed a multi‐step mapping strategy. A distinct list of reported product names in FAERS (533,032 entries) was extracted, including both raw reported names and normalized active ingredient fields. This dataset was saved as a CSV file (FAERS_products.csv) and processed using our FaersMap tool, a component of the PVLens processing pipeline.

The mapping process involved the following steps:

1. Direct string matching of reported or standardized product names to known SNOMED and RxNorm terms.
2. Substring matching for unresolved terms, excluding ambiguous or overly generic ingredients (e.g., “aspirin,” “vitamin”).
3. Active moiety matching using normalized ingredient identifiers.
4. Multi‐step validation to ensure mapped drugs had valid PVLens substance IDs and supporting evidence (e.g., label‐based AE or indication entries).

FAERS products that successfully matched to PVLens substances—based on consistent RxNorm or SNOMED identifiers and verified supporting AE or indication data—were retained. From this group, a final set of 69 drugs was selected using two criteria: (1) availability of safety data following FDA approval, and (2) evidence of new safety label content added within two years of product launch. These criteria ensured the inclusion of products with robust post‐marketing safety data and documented label changes, enabling evaluation of signal detection performance over time.

All AEs within FAERS were coded using the MedDRA version 26.1. DPAs were conducted at the PT level, and SSMs were computed between PTs using the MedDRA hierarchy.

### 2.2 Semantic Similarity Measures

Ontology‐based SSMs are widely used in biomedical informatics but remain underutilized in PV. These measures enable the quantification of relatedness between medical concepts and support more nuanced grouping of AEs beyond the rigid hierarchical structure of terminologies like MedDRA.

SSMs are generally categorized into four main types:

1. *Path‐based*: Based on shortest path lengths between terms in the ontology graph.
2. *Corpus information content (ICont)‐based*: Derived from the frequency of terms in a large corpus of biomedical text.
3. *Intrinsic ICont‐based*: Calculated using only the structure of the ontology, without relying on external corpora.
4. *Vector‐based*: Learned from data, such as co‐occurrence patterns in SRSs.

Among these, intrinsic *ICont*‐based SSMs offer particular advantages for safety surveillance, as they do not require external data sources and are robust to updates in terminologies and databases [49].

In both the corpus *ICont*‐based SSMs and intrinsic *ICont*‐based SSMs, the SSMs are computed from the information content of the concepts. *ICont* represents the degree of informativeness of a given term based on its occurrence within a corpus of text. When corpus‐based, the information content is calculated as ICont(c)=−log(p(c)), where p(c) denotes the probability of concept c within a specified corpus of texts. Conversely, in the intrinsic version, the information content of a concept is determined directly from the structure of the ontology [49]:

$$ICont_{Intrinsic}(c)\approx -\log\left(\frac{\frac{leaves(c)}{subsumers(c)}+1}{tot\_leaves+1}\right)$$

where leaves(c) is the set of concepts found at the end of the ontology under concept c and subsumers(c) is the complete set of ontological ancestors of c including itself. Finally, tot_leaves is the total number of leaves in the ontology.

In our prior evaluation of SSMs for clustering MedDRA PTs in drug safety data, intrinsic *ICont*‐based consistently outperformed path‐based alternatives when measured using F1‐scores against SMQs and expert medical review [50].

For this study, we adopted the Sokal measure—an intrinsic *ICont*‐based metric derived from the hierarchical integration of MedDRA and SNOMED CT via the unified medical language system (UMLS)—as our preferred semantic similarity method [49, 51].

The Sokal SSM between two concepts $c_{1}$ and $c_{2}$ incorporates a specific normalization factor:

$$\begin{array}{ll} & \text{SOKAL}(c_{1},c_{2})\;=\frac{ICont\left(\text{LCA}(c_{1},c_{2})\right)}{2\times \left(ICont(c_{1})+ICont(c_{2})\right)-3\times ICont\left(\text{LCA}(c_{1},c_{2})\right)} \end{array}$$

where $ICont\left(\text{LCA}(c_{1},c_{2})\right)$ is the intrinsic ICont of the lowest common ancestor of the two concepts in the ontology.

Several considerations guided the selection of the Sokal measure:

- It achieved among the highest F1‐scores (0.403) in our feasibility assessment, comparable to intrinsic Lin [52] and intrinsic *ICont*‐based Leacock and Chodorow (LCH) [53].
- Unlike Lin, the Sokal measure assigns more weight to higher similarity values, which is advantageous in Bayesian borrowing frameworks where accurate weighting enhances inference.
- While LCH also performed well, its unbounded scale complicates its use in Bayesian models, which require similarity scores to be normalized between 0 and 1.

Although, several intrinsic *ICont*‐based SSMs demonstrated strong performance relative to path‐based methods, our decision to focus on the Sokal measure was motivated by its favorable balance of discriminative power, interpretability, and compatibility with our BDB framework. Importantly, as an intrinsic *ICont*‐based method, the Sokal measure relies exclusively on the structured relationships encoded in MedDRA and SNOMED CT via UMLS, making it entirely independent of FAERS co‐reporting data.

### 2.3 Statistical Methods

Table 1 presents the contingency table used for DPA of each product‐event pair, where y denotes the presence of the AE of interest in an ICSR, and x indicates the presence of the drug of interest in the same ICSR.

**TABLE 1:** Contingency table for product‐event pairs.

	y	Not y
x	a	b
Not x	c	d

The basic DPA measure used in this study is the relative reporting ratio (RRR), also known as the observed‐to‐expected ratio ($\frac{O}{E}$):

$$\frac{O}{E}=\frac{a}{\frac{\left(a+b)(a+c\right)}{a+b+c+d}}.$$

This ratio compares the observed count a to the expected count under the assumption of independence between the drug and event.

To evaluate DPA in SRSs, we employed the IC analysis within a Bayesian hierarchical framework. This framework allows the incorporation of a robust MAP prior to enable BDB from other PTs based on their semantic similarity to the PT of interest. The IC SSM approach was compared against two alternatives:

1. Traditional IC analysis with no borrowing from other PTs, and
2. IC analysis using BDB from PTs within the same MedDRA HLGT, where equal weight was assigned to each PT included in the prior. Across all methods, a statistical signal was defined as a lower limit of the 95% credible interval (CrI) for the IC exceeding zero. This consistent signal definition allowed us to isolate and assess the added value of semantic similarity‐based borrowing in identifying safety signals.

#### 2.3.1 IC

The IC is defined as the base‐2 logarithm of the ratio of observed to expected joint probabilities of a drug‐event pair, under the assumption of independence [17]:

$$IC=\log_{2}\frac{P(x,y)}{P(x)P(y)}$$

Bayesian inference is used to estimate this quantity. Following the approach of Norén et al. [11], we assume that the cell counts a, b, c, and d follow a multinomial distribution:

$$\left(a,b,c,d)∼\text{Multinomial}(N;p_{a},p_{b},p_{c},p_{d}\right)$$

where N=a+b+c+d is the total number of ICSRs, and $p_{a}$, $p_{b}$, $p_{c}$, and $p_{d}$ represent the probabilities associated with each cell in the contingency table. A Dirichlet prior is placed on these probabilities:

$$\left(p_{a},p_{b},p_{c},p_{d})∼\text{Dirichlet}(\alpha_{a},\alpha_{b},\alpha_{c},\alpha_{d}\right)$$

resulting in a posterior distribution that is also Dirichlet:

$$\text{Dirichlet}(\gamma_{a},\gamma_{b},\gamma_{c},\gamma_{d})\;\text{with}\;\gamma_{i}=\alpha_{i}+i\;\text{for}\;i\in \{a,b,c,d\}$$

This prior moderates the estimated association strength, particularly when data support is weak, helping to stabilize estimates and address zero‐cell issues. It is configured to center the posterior IC distribution between zero and the observed log(O/E), thereby providing a conservative yet robust inference approach.

In this study, posterior estimates of the IC and its CrI were derived from Monte Carlo simulations. As the IC distribution is typically unimodal, the posterior mean estimate (PME) was used as the central value. Although, Norén recommends using the maximum a posteriori estimate, we opted for the PME due to its conservative nature. This choice has limited influence on signal detection when alerts are defined by the CrI lower limit but is important in the context of Bayesian borrowing, where both the central IC and its variance are used to inform the prior.

All IC calculations were performed using the publicly available BCPNN R function^2^ [21].

#### 2.3.2 Robust MAP Prior for BDB Based on Semantic Similarity Between Outcomes

The IC SSM method is inspired by dynamic borrowing techniques used in clinical trials, particularly the robust MAP prior framework [42, 44]. In our context, we adapt this approach for PV by dynamically borrowing information from other AEs reported with the same product. Specifically, for each product‐event pair of interest, we construct a robustified MAP prior using a weighted combination of IC estimates from similar events, leveraging SSMs between MedDRA PTs.

Let $Y_{I}$ and $IC_{I}$ denote the data and IC for the product‐event pair of interest, with $Y_{I}$ data comprising the elements of the contingency Table 1. Let $Y_{B}=(Y_{1},\ldots ,Y_{S})$ and $IC_{B}=(IC_{1},\ldots ,IC_{S})$ represent the data and IC values of S semantically similar events, and let $SSM_{B}=(SSM_{1},\ldots ,SSM_{S})$ denote their corresponding similarity scores, where each $SSM_{s}\in [0,1]$, with 0 indicating no similarity and 1 indicating synonymy. Inference on $IC_{I}$ is based on both direct evidence from $Y_{I}$ and indirect evidence from $\left(Y_{B},SSM_{B}\right)$, implemented in two steps. First, a MAP prior $p(IC_{I}|Y_{B},SSM_{B})$ is constructed. Then, this prior is updated via Bayes' theorem:

$$p(IC_{I}|Y_{I},Y_{B},SSM_{B})\propto p(Y_{I}|IC_{I})\cdot p(IC_{I}|Y_{B},SSM_{B})$$

Assuming each estimated ${\hat{\text{IC}}}_{s}$ follows a normal distribution centered on its true value and with variance ${\text{VIC}}_{s}$:

$${\hat{\text{IC}}}_{s}|{\text{IC}}_{s},{\text{VIC}}_{s}∼𝒩({\text{IC}}_{s},{\text{VIC}}_{s}),\;s=1,\ldots ,S$$

we account for heterogeneity among similar events using a random‐effects framework:

$$IC_{I},IC_{1},\ldots ,IC_{S}∼𝒩(\mu ,\tau^{2})$$

Here, μ is the population mean and τ the between‐event standard deviation.

In standard MAP estimation, IC values are typically weighted by the inverse of their variances. In our adaptation, these weights are further multiplied by semantic similarity to reflect both statistical uncertainty (via variance) and clinical relevance (via semantic similarity). This approach is analogous to meta‐analytic techniques that incorporate study quality into weighting schemes [54].

To illustrate the role of SSMs in the weighting process, we first present the fixed‐effect meta‐analytic formulation of the MAP prior:

$${\hat{p}}_{B}({\text{IC}}_{I})∼𝒩({\hat{\mu }}_{m},{\hat{V}}_{m})$$

with the weighted mean and variance defined as:

$${\hat{\mu }}_{m}=\frac{\sum_{s}\left(\frac{{\text{SSM}}_{s}}{{\text{VIC}}_{s}}\cdot {\text{IC}}_{s}\right)}{\sum_{s}\frac{{\text{SSM}}_{s}}{{\text{VIC}}_{s}}}\;\text{and}\;{\hat{V}}_{m}=\frac{\sum_{s}\frac{{\text{SSM}}_{s}^{2}}{{\text{VIC}}_{s}}}{{\left(\sum_{s}\frac{{\text{SSM}}_{s}}{{\text{VIC}}_{s}}\right)}^{2}}$$

Although, this fixed‐effect formulation clarifies how SSMs are incorporated, our implementation used a random‐effects meta‐analysis to accommodate heterogeneity. MAP priors were estimated using restricted maximum likelihood via the rma() function in the R package metafor [55].

Even when SSMs are derived from structured and clinically meaningful ontologies, the assumption of exchangeability among the borrowed PTs may not fully hold. Such violations can introduce prior‐data conflict. To mitigate this, we adopt a robust version of the MAP prior:

$${\hat{p}}_{\text{BR}}({\text{IC}}_{I})=w\cdot {\hat{p}}_{B}({\text{IC}}_{I})+(1-w)\cdot p_{V}({\text{IC}}_{I})$$

where ${\hat{p}}_{B}({\text{IC}}_{I})$ is the MAP prior from the meta‐analysis, and $p_{V}({\text{IC}}_{I})$ is a vague normal prior centered at zero, representing the null hypothesis of no association. This formulation provides robustness in the event of prior‐data conflict, meaning when the IC of the event of interest diverges meaningfully from those of similar events, even after accounting for their weighted contributions in the prior. It aligns with Bayesian DPA strategies that emphasize shrinkage toward zero in the presence of weak evidence [7, 10, 11, 17].

Following Schmidli et al. [44], the vague prior was based on the log‐transformed ratio of a binomial proportion with a Beta(1,1) prior. The resulting vague prior for the IC is expressed as:

$$\log_{2}\left(\frac{\text{Beta}(1,1)}{\text{Beta}(1,1)}\right)$$

which corresponds to a standard deviation (σ) of approximately 2.0, capturing substantial uncertainty.

While w could be fixed arbitrarily (e.g., w=0.8), such an approach fails to account for the level of similarity between the PT of interest and the PTs used for borrowing. Importantly, even if relative similarities are incorporated in the MAP prior, a fixed w does not distinguish between borrowing from PTs with uniformly low similarity and borrowing where at least one PT has a similarity score near 0.95.

To reflect this nuance, we link w to the maximum observed similarity $\max(SSM_{B})$, so that borrowing is more substantial when at least one PT is highly similar. If a single PT has strong similarity to the PT of interest, it should dominate the MAP prior—even if other borrowed terms are less related. In contrast, if all SSMs are low, borrowing should be limited. Using the maximum rather than the mean or median ensures that w reflects the strongest available similarity.

Prior work has shown that the recall of PT clustering was barely improved when including PTs with SSM below 0.3, as compared to clinical expert judgment [50]. Therefore, while removing this threshold of inclusion would not necessarily pose an issue, the added value of including PTs with SSM below 0.3 was anticipated to have limited impact on the MAP prior. Consequently, only PTs with an SSM greater than 0.3 are included for borrowing in the MAP prior, which also enhances computational efficiency in large‐scale DPA.

The resulting robust prior is a mixture of two conjugate normal distributions:

$${\hat{p}}_{\text{BR}}({\text{IC}}_{I})=w\cdot 𝒩({\text{IC}}_{I}|{\hat{\text{IC}}}_{B},{\hat{\text{VIC}}}_{B})+(1-w)\cdot 𝒩({\text{IC}}_{I}|0,2^{2})$$

and the posterior distribution, also a mixture, is given by:

$$\begin{array}{ll} {\hat{p}}_{\text{BR}}({\text{IC}}_{I}|Y_{I})\; & =\tilde{w}\cdot 𝒩\left({\text{IC}}_{I}\;|\;\frac{{\hat{\text{IC}}}_{B}\;{\hat{\text{VIC}}}_{B}^{-1}+{\hat{\text{IC}}}_{I}\;{\hat{\text{VIC}}}_{I}^{-1}}{{\hat{\text{VIC}}}_{B}^{-1}+{\hat{\text{VIC}}}_{I}^{-1}},\frac{1}{{\hat{\text{VIC}}}_{B}^{-1}+{\hat{\text{VIC}}}_{I}^{-1}}\right)\\ & \;+(1-\tilde{w})\cdot 𝒩\left({\text{IC}}_{I}\;|\;\frac{{\hat{\text{IC}}}_{I}\;{\hat{\text{VIC}}}_{I}^{-1}}{4^{-1}+{\hat{\text{VIC}}}_{I}^{-1}},\frac{1}{4^{-1}+{\hat{\text{VIC}}}_{I}^{-1}}\right) \end{array}$$

where $\tilde{w}$ is the updated posterior mixture weight, computed according to Best et al. [56].

#### 2.3.3 Robust MAP Prior for BDB Based on MedDRA HLGT

In addition to the semantically informed borrowing in IC SSM, we implemented an alternative approach—termed IC HLGT—which leverages BDB constrained to the MedDRA HLGT. While the overall borrowing framework is similar to that used in IC SSM, there are three primary distinctions in the implementation of IC HLGT:

- Selection of borrowing terms: In IC HLGT, only PTs within the same HLGT as the target PT are included in the MAP prior. In contrast, the IC SSM approach can potentially include all PTs, provided computational resources allow, but in this study only those with a Sokal SSM greater than 0.3 were considered.
- Determination of weights within the MAP prior: For IC HLGT, the weights assigned to each PT within the MAP prior are based solely on the statistical uncertainty—specifically, on inverse of the variance of the traditional IC estimates—following a standard meta‐analytic framework. This contrasts with the IC SSM approach, where weights also incorporate clinical similarity (via the SSM).
- Overall weight of the MAP prior in the robust mixture prior: In the construction of the robust mixture prior, IC SSM adjusts the weight (denoted as w) assigned to the MAP prior based on the maximum SSM of the selected PTs. In IC HLGT, by contrast, this overall weight w is fixed at 0.8, meaning that the borrowing strength is constant regardless of the degree of clinical similarity within the HLGT.

This HLGT‐based implementation serves as a benchmark for assessing the added value of incorporating semantic similarity into BDB, allowing us to evaluate how clinical context can influence signal detection beyond what is achieved through variance‐based weighting alone in predefined groupings of PTs.

### 2.4 Reference Set

Robust evaluation of signal detection methods in PV requires high‐quality references sets [47]. Norén et al. [57] emphasized the importance of assessing methods using emerging rather than established adverse drug reactions, underscoring the need for time‐stamped reference standards that reflect the state of knowledge before a given label change [57]. While several initiatives have attempted to construct large‐scale reference sets over the past decade, including those based on electronic health records or spontaneous reports, consistent quality and temporal alignment have remained elusive [58].

To address this gap, we utilized a newly developed reference set generated using PVLens, an automated system that extracts indications, black box warnings, and AE terms from FDA SPL documents and maps them to MedDRA [47]. PVLens integrates RxNorm, SNOMED CT, and the UMLS MTHSPL tables to align AE terms with their respective substances and track label changes over time. Its automated natural language processing pipeline ensures high recall of labeled safety events, and expert adjudication has demonstrated reliable precision in its ability to map and assign MedDRA terms.

For this study, we constructed a positive control set consisting of MedDRA PTs added to US product labels between 2016 and 2019, capturing newly labeled safety information after initial FDA approval. These terms were extracted directly from SPL change logs and verified against historical label versions. By anchoring each AE to its date of labeling, we ensured temporal separation between the data used for signal detection (2015–2019 FAERS) and the subsequent inclusion of that AE in the label, enabling a prospective evaluation of method performance and mitigating reporting bias.

For the negative control set, we followed the guidance of Norén et al. by including all PTs that did not share the same MedDRA high‐level term (HLT) as any of the positive control PTs, regardless of whether they appeared in historical labeling [57]. This conservative definition helps reduce the risk of inadvertently labeling true signals as negatives while maintaining a realistic class imbalance that reflects the distribution of potential safety signals in real‐world data.

By leveraging PVLens as a validated (although not perfect), time‐aware reference source, this approach improves upon past methods by enabling reproducible, expert‐informed evaluations of safety signal detection across dynamic PV datasets.

### 2.5 Performance Evaluations

The evaluation of method performance was conducted in multiple stages: A pre‐specified primary analysis comparing the proposed approach to a current reference standard, as well as a previously published approach to Bayesian borrowing, all with the standard operational implementation parameters, followed by a set of sensitivity analyses to assess the model's robustness to its key parameters, and finally, a series of post hoc exploratory analyses designed to investigate specific findings that emerged from the primary results.

#### 2.5.1 Primary Performance Analysis

The primary analysis compared IC SSM against traditional IC and IC HLGT using a statistical signal defined as the lower limit of the 95% CrI for the IC exceeding zero. This corresponds to the conventional 2.5% alerting quantile employed by the Uppsala Monitoring Centre [59].

Performance evaluations were conducted on a quarterly basis from January 1, 2016, to December 31, 2019. For each quarter, we computed sensitivity (i.e., recall), specificity, positive predictive value (PPV) (i.e., precision), Youden's index, and F1‐score using the data accumulated since January 1, 2015, up to that point.

Performance metrics were also summarized across the entire analysis period.

True positives (TPs) were defined as positive controls alerted before the label update, with positive controls alerted after the label update being ignored; false positives (FPs) were negative controls alerted at any time; true negatives (TNs) were negative controls never alerted; and false negatives (FNs) were positive controls never alerted before the label update.

Descriptive analyses were conducted to compare the performance of two methods using a one‐to‐one comparison framework. Specifically, we categorized the detection outcomes to detail the number of TPs uniquely identified by each method, the number of TPs concurrently detected by both methods, and the number of TPs that were not detected by either method. Furthermore, we examined the differences in the time‐to‐detection of the TPs. For TPs detected by only one of the two methods, 2020Q1 was imputed as the quarter of detection.

#### 2.5.2 Parameter Sensitivity Analysis

The performance of the IC SSM is contingent upon three key parameters: The prior value of the weight of the MAP prior (w) in the robust mixture prior, the standard deviation of the vague prior (σ), and the lower limit of the SSM (minSSM) for PTs to be incorporated into the MAP prior. The uncertainty about the best combination of these parameters led us to perform some sensitivity analysis. To evaluate the influence of these parameters, we conducted a series of analyses varying one parameter at a time, keeping the other two as in a reference parametrization with σ=2.0, w=max(SSM), and minSSM=0.3. The impact of varying σ and w on IC HLGT was also evaluated.

#### 2.5.3 Post Hoc Exploratory Analyses

The primary analyses suggested that IC SSM was operating on a different region of the precision‐recall curve and that its advantages were more pronounced in the early surveillance period. To investigate these observations in a structured manner, a series of post hoc exploratory analyses were conducted.

- To explore the trade‐off between sensitivity and precision, particularly in scenarios where precision is prioritized, an analysis was conducted by varying the alerting threshold itself (i.e., the value the lower bound of the 95% CrI must exceed), from 0 up to 1.0.
- To further explore the sensitivity‐precision trade‐off, additional analyses were run by varying the alerting quantiles. IC SSM was evaluated at stricter quantiles of 1% (IC SSM q0.01), 1.5%, and 2%. To create a precision‐matched comparison, traditional IC was evaluated at a relaxed 5% quantile (IC q0.05).
- To test the hypothesis that the method's advantage was concentrated in early surveillance, performance was stratified by the cumulative number of cases for each product‐event pair. Metrics were computed for subsets of pairs with ≤3, ≤4, and ≤5 cases.
- To determine if the observed timeliness advantage was driven by performance in sparse‐data settings, the time‐to‐detection analysis was stratified. The distribution of the difference in time‐to‐detection between IC SSM and traditional IC was re‐evaluated, separating TPs first detected when the product‐event pair had five or fewer cumulative cases versus more than five cases.

## 3 Results

### 3.1 Temporal Trends in Method Performance

The performance of the proposed IC SSM method was evaluated over time to assess its practical value in dynamic post‐marketing surveillance scenarios. IC SSM demonstrated clear advantages in sensitivity and early detection—two key priorities in PV, where timely hypothesis generation can allow for appropriate communication of risk.

During the study period, the number of distinct product‐event pairs reported per quarter increased markedly, from 3474 in 2016Q1 to 30 589 in 2019Q4, reflecting the growing volume and breadth of safety data. In parallel, the number of positive controls in the reference set expanded from 603 to 1948. However, the proportion of positives decreased over time due to the removal of events once added to product labels. The trends in the number of alerts and TPs identified by each method over the analysis period are illustrated in Supporting Information Figure S1. By Q4 2019, the traditional IC, IC SSM, and IC HLGT methods generated 6112, 7386, and 7525 alerts, respectively. The figure also includes the curve for IC SSM when the alerting quantile is decreased to 1%, achieving more comparable precision with traditional IC. The curve was mainly positioned on the upper left side of the traditional IC curve, indicating a lower number of alerts for a higher number of TPs; notably, under this setting, IC SSM q0.01 generated 6013 alerts by Q4 2019—fewer than traditional IC.

Figure 1 shows the evolution of sensitivity and PPV for IC SSM and the two benchmarking methods during the analysis period. Overall, traditional IC exhibited a higher PPV than both IC SSM and IC HLGT throughout the study period, with the PPV of IC SSM and IC HLGT remaining very similar. For all three methods, PPV decreased over time, and the proportional difference between traditional IC and the other methods remained relatively constant. In terms of sensitivity, traditional IC showed a slight increase over time, rising from 42.6% in 2016Q1 to 44.0% in 2019Q4. By contrast, IC SSM began with a higher sensitivity of 53.6% in 2016Q1 and, although it decreased to 47.9% by 2019Q4, it consistently maintained a sensitivity superior to that of traditional IC. Additionally, IC SSM consistently outperformed IC HLGT in terms of sensitivity, although the differences were less pronounced compared to those observed with traditional IC. These results underscore the strength of the IC SSM method in early signal detection compared to traditional IC. Figure 1 also includes curves for two exploratory configurations designed to enable more direct performance comparisons: IC SSM with a stricter alerting quantile (IC SSM q0.01) and traditional IC with a relaxed quantile (IC q0.05). The stricter IC SSM configuration (IC SSM q0.01) demonstrates that the method can achieve slightly higher precision than traditional IC while still maintaining substantially higher sensitivity. Conversely, the relaxed traditional IC configuration (IC q0.05) shows that even when tuned for comparable precision to the default IC SSM, its sensitivity remains lower. This advantage in sensitivity for both IC SSM configurations is especially pronounced during the early phases of the analysis period, when early signal detection is most valuable following marketing authorization.

**FIGURE 1:**
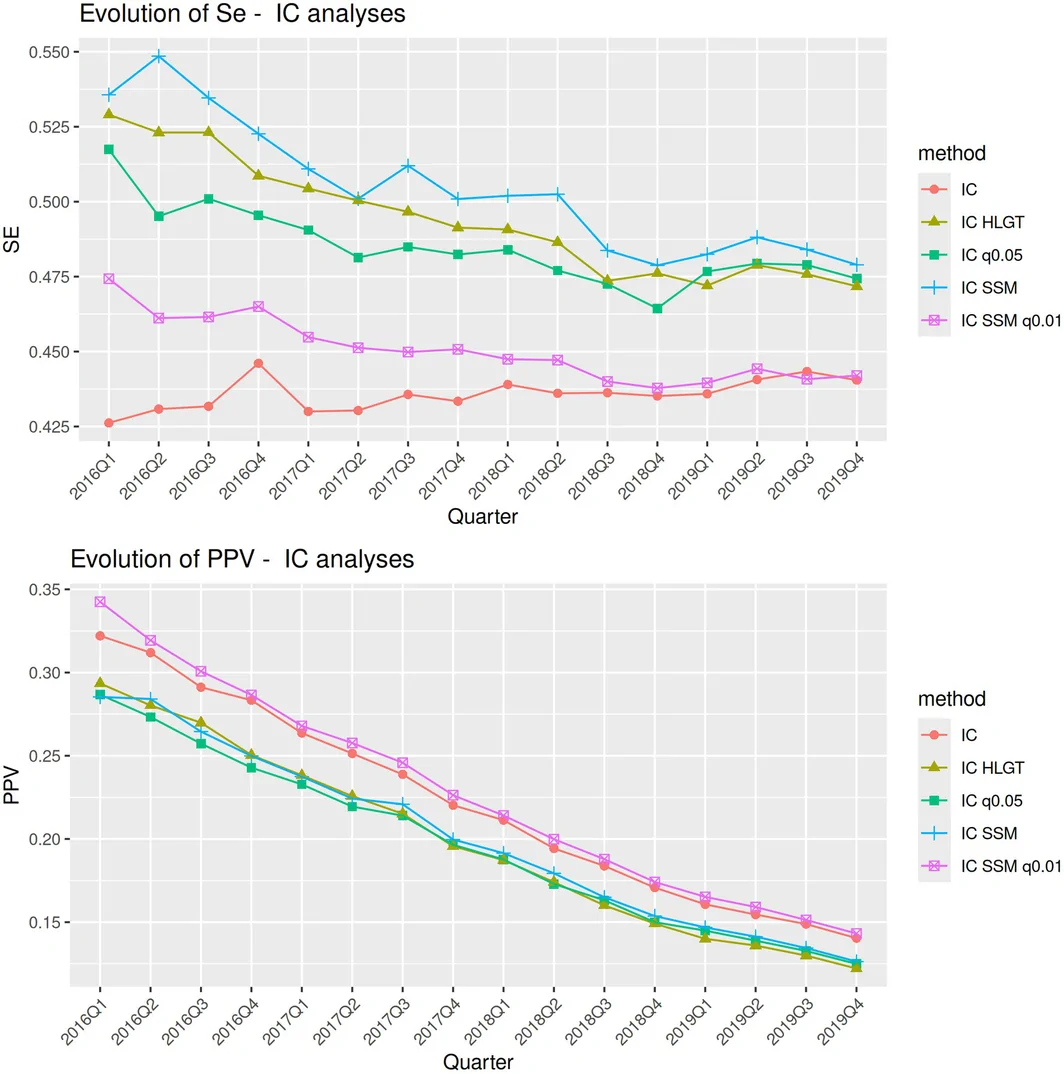
Evolution over time of the sensitivity (SE) and positive predictive value (PPV). IC SSM q0.01 means IC SSM method with alerting quantile of 1%—corresponding to the lower limit of the 98% CrI >0. When not mentioned, the standard 2.5% quantile was used.

### 3.2 Performance Analyses Over the Entire Study Period

#### 3.2.1 Primary Performance Analyses

Performance analyses over the entire study period are presented in Figure 2.

**FIGURE 2:**
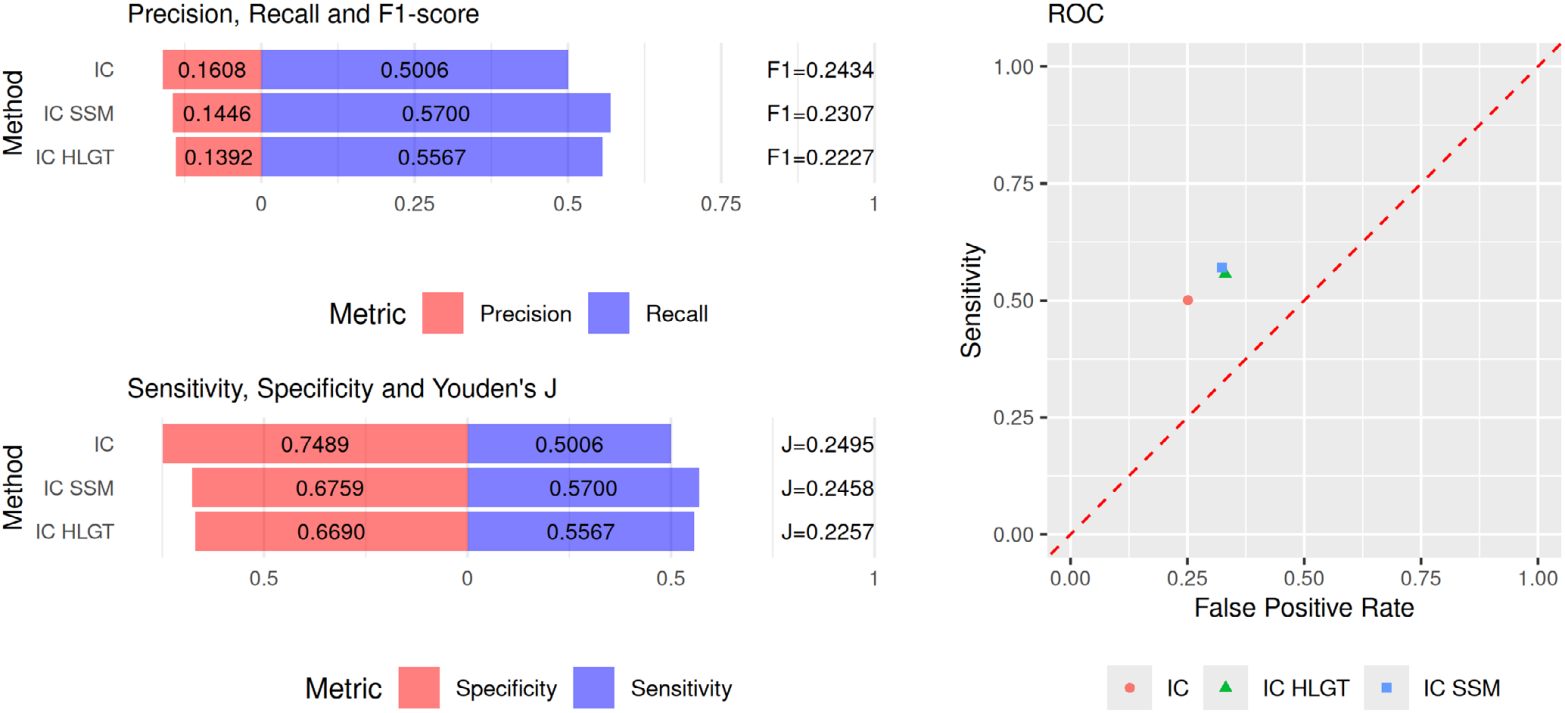
Primary performance analyses over the entire study period. IC = Traditional IC.

When evaluated at their conventional alerting thresholds, the results revealed important differences between the methods. The sensitivity of traditional IC analysis was 50.1%, whereas IC SSM exhibited a higher sensitivity of 57.0%. This increase in sensitivity was accompanied by a decrease in precision, from 16.1% to 14.5%, resulting in a decrease in the F1 score from 0.243 to 0.231. Additionally, specificity decreased from 74.9% with IC analysis to 67.6% with IC SSM. The resulting Youden's index was slightly lower for IC SSM than for IC without borrowing, with 0.246 and 0.250, respectively. When comparing IC HLGT to IC SSM, the differences were less pronounced than those between IC and IC SSM. However, IC HLGT exhibited lower sensitivity, specificity, and precision, resulting in a lower F1 score (0.223) and Youden's index (0.226).

Two‐by‐two comparisons between methods are summarized in Table 2 and Figures 3 and 4. These comparisons focus on the absolute numbers of TPs that were concordant and discordant between each method, providing a more detailed view of their relative performance. IC SSM detected 165 positive controls that IC analysis did not identify, while IC analysis detected three positive controls that IC SSM missed. Among the 1167 positive controls detected by both methods, 817 were identified within the same calendar quarter.

**FIGURE 3:**
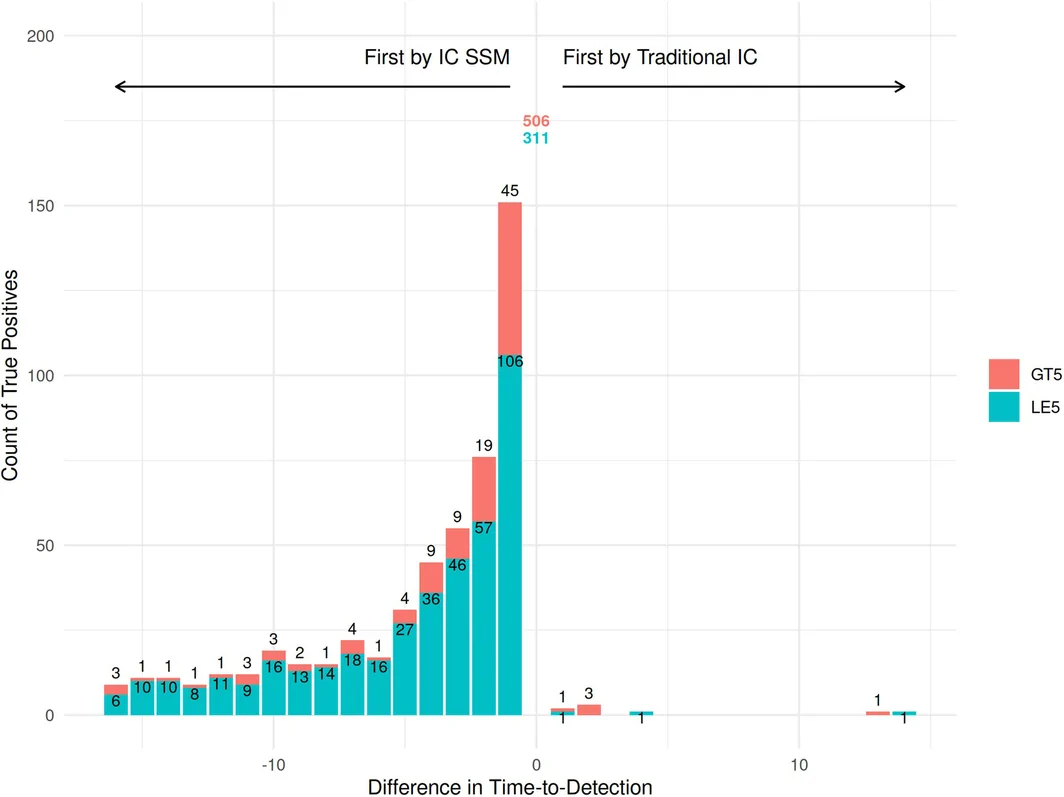
Distribution of the difference in time‐to‐detection of true positives between IC SSM and IC. 817 TPs were detected in the same quarter by both methods. IC = Traditional IC, GT5 = product‐event pairs with more than five cases at first alert, LE5 = product‐event pairs with five cases or less at first alert.

**FIGURE 4:**
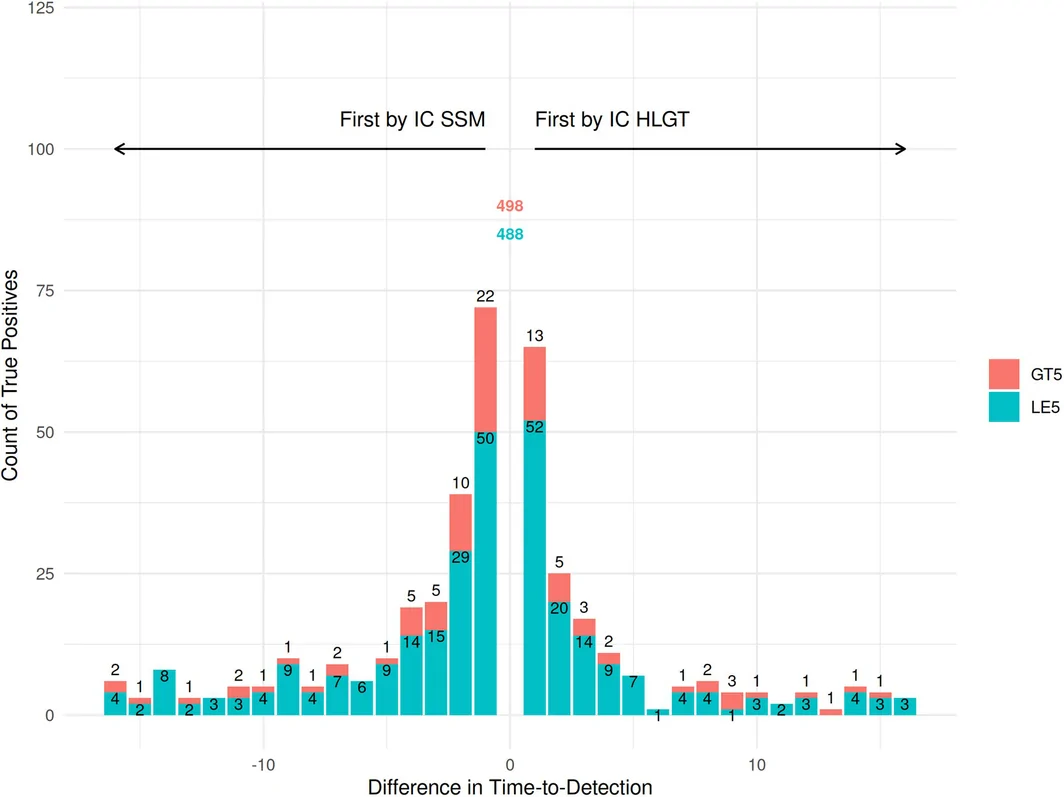
Distribution of the difference in time‐to‐detection of true positives between IC SSM and IC HLGT. GT5 = product‐event pairs with more than five cases at first alert, LE5 = product‐event pairs with five cases or less at first alert.

**TABLE 2:** Two‐by‐two contingency tables comparing IC SSM with IC and IC HLGT for detecting positive controls.

	IC			IC HLGT	
IC SSM	+	−		+	−
+	1167	165		1260	72
−	3	1002		41	964

A key performance differentiator was the time‐to‐detection. For the primary comparison, the distribution of differences in time‐to‐detection of positive controls is depicted in Figure 3, illustrating that IC SSM generally detected more positive controls and earlier than IC analysis, with an average difference of 1.75 quarters. When comparing IC SSM with IC HLGT, IC SSM identified 72 positive controls that IC HLGT did not detect, while IC HLGT found 41 positive controls that IC SSM missed. Of the 1260 positive controls detected by both methods, 986 were detected in the same calendar quarter. The distribution of differences in time‐to‐detection of positive controls favored IC SSM with an average difference of 0.24 quarters (Figure 4).

We assessed the robustness of IC SSM's superior performance across variations in the reference set by employing a resampling method. One thousand new reference sets of equivalent size were generated through random sampling with replacement, and performance metrics were computed for each set. The results indicated that IC SSM demonstrated higher sensitivity compared to IC HLGT in 99.9% of the simulations. Furthermore, IC SSM exhibited superior performance in terms of Youden's index, specificity, and F1 score in 100% of the simulations when compared to IC HLGT. Additionally, IC SSM achieved a lower F1 score than the IC without Bayesian borrowing in 100% of the simulations, and it demonstrated a higher Youden's index than the IC without Bayesian borrowing in 24.2% of the simulations.

#### 3.2.2 Post Hoc Exploratory Analyses

The following analyses were conducted to explore the trade‐offs and early‐stage performance trends suggested by the primary results.

To enhance precision and specificity, particularly when resource constraints limit the number of signals that can be reviewed, the threshold for the lower limit of the 95% CrI can be increased. As shown in Supporting Information Figure S2, this adjustment improved precision and specificity for IC SSM, whereas performance for traditional IC declined. At a threshold of 1, IC SSM achieved an F1 score of 0.239 and Youden's index of 0.191, compared with 0.236 and 0.182, respectively, for traditional IC. Although, this threshold increase did not raise IC SSM's F1 score above that of traditional IC at its regular threshold (i.e., 0), it enhanced overall precision and specificity for IC SSM.

Alternatively, precision and specificity can be adjusted by changing the alerting quantile of the posterior IC distribution, with the results summarized in Supporting Information Figure S3. To facilitate a comparison at a similar level of precision, an exploratory analysis evaluated IC SSM with a stricter alerting quantile of 1% (IC SSM q0.01). At this setting, the precision and specificity of IC SSM were similar to those of traditional IC (16.4% vs. 16.1% and 74.8% vs. 74.9%, respectively), while IC SSM maintained higher sensitivity (51.4% vs. 50.1%) and also yielded the highest F1 score (0.249) and Youden's index (0.262) relative to both traditional IC and IC HLGT. Conversely, a second exploratory analysis assessed if traditional IC could match the sensitivity of the primary IC SSM configuration. Using a wider 5% alerting quantile (IC q0.05), the sensitivity of traditional IC increased to 55.6%, though it remained lower than the 57.0% observed with IC SSM. The precision of IC q0.05 concurrently decreased to 14.2%, a level comparable to that of IC SSM (14.5%). These exploratory findings suggest that the performance profile of IC SSM may not be readily replicated by the traditional IC method through simple quantile adjustment.

To formally test the hypothesis that the method's advantage was concentrated in early surveillance, performance was stratified by the cumulative number of reported cases. As presented in Table 3, IC SSM showed a clear performance advantage in low‐count scenarios, especially when compared directly with traditional IC. For product‐event pairs with three or fewer cases, IC SSM achieved a substantially higher F1‐score than traditional IC (0.141 vs. 0.098), driven by a more than two‐fold increase in sensitivity (0.260 vs. 0.109) and an improvement in precision (0.096 vs. 0.089). It also outperformed the relaxed IC q0.05 and IC HLGT configurations across all metrics in this stratum. This demonstrates that the superior F1‐score of IC SSM in early surveillance is driven by a notable gain in sensitivity without a sacrifice in precision.

**TABLE 3:** Performance metrics stratified by cumulative case count.

Method	Case count ≤3	Case count ≤4	Case count ≤5
IC SSM q0.025	0.141 (0.260; 0.096)	0.160 (0.326; 0.106)	0.169 (0.365; 0.110)
IC HLGT q0.025	0.129 (0.248; 0.087)	0.150 (0.313; 0.099)	0.159 (0.351; 0.103)
IC q0.05	0.117 (0.201; 0.083)	0.144 (0.281; 0.097)	0.155 (0.324; 0.101)
IC q0.025	0.098 (0.109; 0.089)	0.138 (0.193; 0.107)	0.155 (0.243; 0.114)

*Note:* Values are presented as F1‐score (recall/sensitivity; precision). IC SSM q0.025 = IC SSM alerting with quantile of 2.5%, IC HLGT = Traditional IC with HLGT alerting with quantile 2.5%, IC q0.05 = Traditional IC alerting with quantile of 5% and IC q0.025 = Traditional IC with the standard 2.5% quantile applied.

The advantage in timeliness observed in the primary analysis was further investigated. First, its consistency was assessed across the other exploratory comparisons. The time‐to‐detection distribution continued to favor IC SSM q0.01 when compared to traditional IC (Supporting Information Figure S4). Furthermore, when compared against the more sensitive traditional IC q0.05 configuration, the primary IC SSM method still detected signals earlier, with an average advantage of 0.39 calendar quarters (Supporting Information Figure S5). Second, to determine if this consistent advantage was driven by performance in sparse‐data scenarios, the time‐to‐detection analysis was stratified. As shown in Figure 3, the analysis revealed that most TPs detected earlier by IC SSM occurred in product‐event pairs with five or fewer cumulative cases at the time of first alert. This suggests that the overall timeliness advantage is primarily driven by the method's enhanced performance in sparse‐data settings.

### 3.3 Parameter Sensitivity Analysis

The performance of IC SSM is influenced by three main parameters: The prior value of the weight of the MAP prior (w), the standard deviation of the vague prior (σ), and the lower limit of SSM (minSSM) for PTs to be included in the MAP prior according to SSM.

Supporting Information Figure S6 shows the performance metrics of IC SSM for varying minSSM thresholds, with w and σ kept constant. Thresholds of 0.1–0.6 were considered. Sensitivity increased from 56.0% to 57.0% as the threshold decreased from 0.6 to 0.4, with specificity decreasing from 68.8% to 67.8%. The highest F1 score (0.233) and Youden's index (0.249) were achieved with thresholds of 0.6 and 0.4, respectively. IC SSM's F1 scores and Youden's indexes were lower than IC without borrowing but higher than IC HLGT.

Supporting Information Figure S7 summarizes the impact of varying w from 70% to 90%. Sensitivity increased while specificity and precision decreased with increasing w for both IC SSM and IC HLGT. The highest Youden's index for IC SSM was 0.239 at w=80%. IC SSM's F1‐score and Youden's index were superior to IC HLGT and inferior to IC.

Supporting Information Figure S8 summarizes the impact of varying σ from 0.5 to 10. Sensitivity increased while precision and specificity decreased with increasing σ for both IC SSM and IC HLGT. The highest Youden's index for IC SSM was 0.258 at σ=0.5, followed by 0.253 at σ=1, and the traditional IC positioned itself between IC SSM with σ=1 and σ=2. IC SSM demonstrated higher F1 scores and Youden's indexes than IC HLGT for the same σ values, with differences in sensitivity increasing and differences in specificity decreasing with increasing σ.

### 3.4 Illustrative Examples of Product‐Event Pairs

Figures 5, 6, 7, 8 present four illustrative examples of distinct product‐event pairs^3^: Two cases in which the IC SSM method outperformed both the traditional IC analysis and IC HLGT in detecting AE signals, and two cases where the IC SSM method generated false positive signals.

**FIGURE 5:**
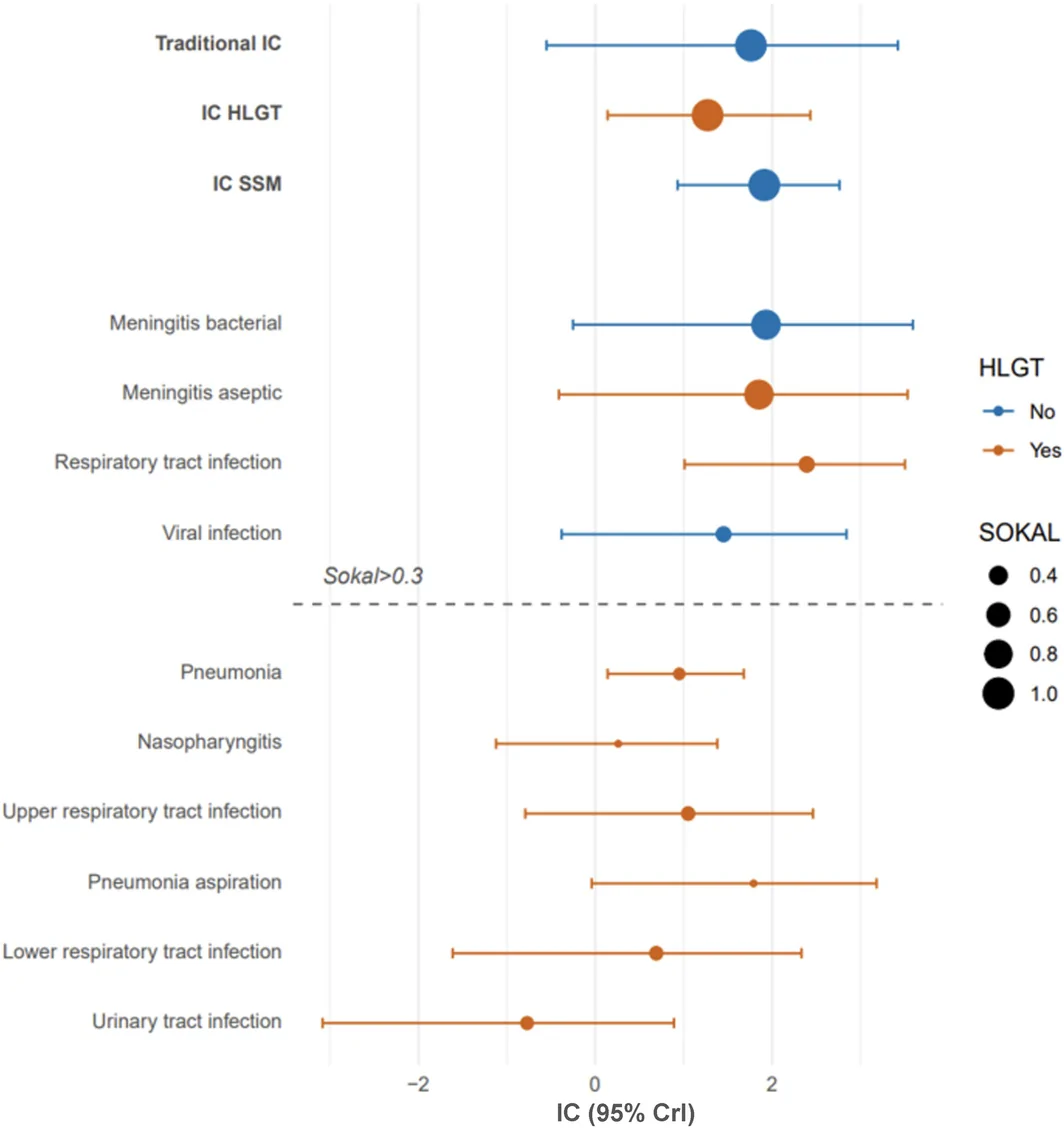
Graphical representation of PTs contributing to Bayesian borrowing in IC SSM and IC HLGT for Example 1: *Meningitis* (2017Q3). Error bars represent the 95% CrIs. The bubble size of the point estimates is proportional to the Sokal similarity with the PT of interest.

#### 3.4.1 Example 1: Meningitis

In this example, the posterior medians of all three methods—IC SSM, IC HLGT, and traditional IC—converged as more data accrued over time. However, IC HLGT consistently lagged behind the other two methods in magnitude. By 2019Q1, IC SSM provided higher and more stable point estimates compared to the other approaches. Moreover, IC SSM consistently demonstrated narrower 95% CrIs, with lower limits remaining above zero across all quarters. Notably, IC SSM alerted for this AE three quarters earlier (2017Q3) than the traditional IC method. In contrast, the lower limit of IC HLGT dropped below zero during the second and third quarters, following an initially positive value.

Figure 5 illustrates the CrIs of IC estimated by the three methods, along with the IC estimates of all the PTs included in the MAP priors of IC SSM and IC HLGT in quarter 2017Q3. The HLGT category for the PT *Meningitis* (i.e., *Infections—pathogen unspecified*), is broad. Examination of other PTs contributing to IC HLGT revealed that several terms within the same HLGT, such as *Pneumonia*, *Nasopharyngitis*, and *Respiratory tract infection*, introduced bias and diluted the signal. In contrast, the SSM‐weighted borrowing in IC SSM prioritized more clinically relevant PTs, including *Meningitis bacterial* (Sokal = 0.87) and *Meningitis aseptic* (Sokal = 0.86), which contributed more accurately to the signal. Notably, while *Meningitis aseptic* was also part of the prior in IC HLGT, its relative weight in IC SSM was significantly higher compared to other PTs, driven by its strong semantic similarity (as indicated by the size of the bubble).

#### 3.4.2 Example 2: Mouth Ulceration

In this example, the IC SSM method consistently produced higher point estimates and higher lower credible bounds compared to both the traditional IC and IC HLGT approaches. IC SSM identified the AE five quarters earlier than IC HLGT and approximately 2.5 years sooner than the traditional IC method.

Figure 6 presents the data for 2017Q1. Within the HLGT *Oral soft tissue conditions*, the most influential MedDRA PT in the MAP prior under IC HLGT was *Stomatitis*, which contributed 61 cases by 2017Q1, followed by *Oral pain* with 38 cases. While both terms are clinically relevant to *Mouth ulceration*, they are broader and less specific. Several other PTs in the same HLGT contributed to the IC HLGT analysis, such as *Oral discomfort* with 11 cases and several others with three or fewer cases. These PTs exhibited wide CrIs that largely overlapped with the CrI of the traditional IC of *Mouth ulceration*. The two most influential PTs in the MAP prior of IC HLGT had a data conflict with the PT of interest *(Mouth ulceration)*, as evidenced by their substantially higher CrIs compared to the CrI of the traditional IC. Consequently, the BDB likely assigned less weight to the MAP prior, thereby decreasing the borrowing effect.

**FIGURE 6:**
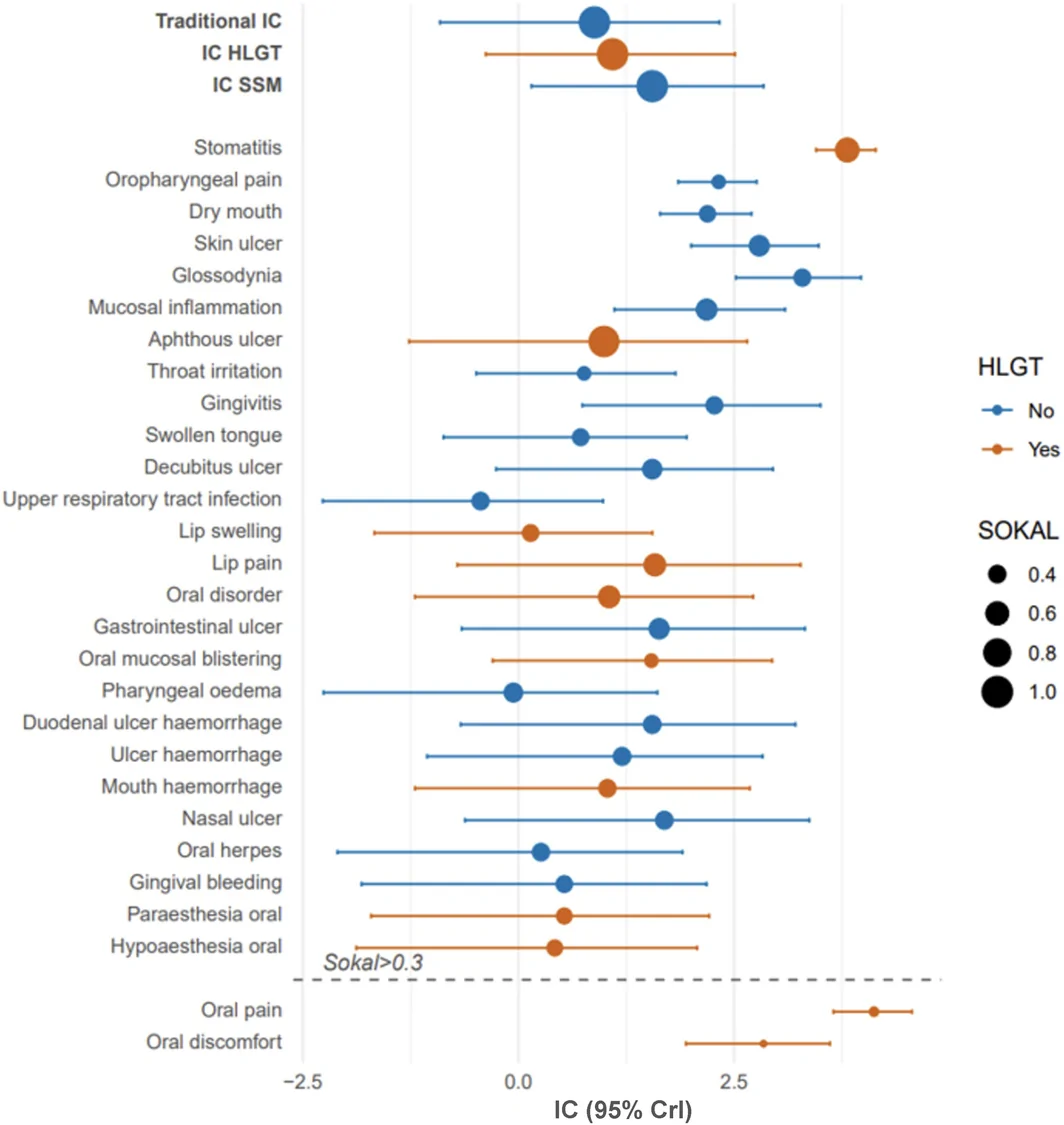
Graphical representation of PTs contributing to Bayesian borrowing in IC SSM and IC HLGT for Example 2: *Mouth ulceration* (2017Q1). Error bars represent the 95% CrIs. The bubble size of the point estimates is proportional to the Sokal similarity with the PT of interest.

In the IC SSM model, *Stomatitis* also carried the greatest weight, but its influence was moderated due to a Sokal semantic similarity score of 0.63—only moderately close to *Mouth ulceration*. Four other PTs (*Oropharyngeal pain, Dry mouth, Skin ulcer*, and *Glossodynia*) with more than 10 cases were among the most influential contributors to the MAP prior, and these were part of other HLGTs. All of them exhibited moderately higher IC estimates than *Mouth ulceration*. Many other PTs with Sokal scores above 0.3 exhibited IC values more consistent with *Mouth ulceration*, collectively contributing to a more targeted and effective borrowing structure. The PT with the highest similarity was *Aphthous ulcer*, with a Sokal similarity of 0.96.

These results highlight how the IC SSM method selectively draws information from clinically similar terms across HLGT boundaries. Additionally, the PTs included in the MAP prior of IC SSM exhibited 95% CrIs of IC that were more consistent with *Mouth ulceration*, leading to a higher weight of the MAP prior in the results.

#### 3.4.3 Example 3: Pharyngeal Erythema

The results for this illustrative example are presented in Figure 7. This case highlights a false positive signal generated by the IC SSM method, which was not identified by the benchmarking methods—the traditional IC and IC HLGT—potentially revealing a limitation of the IC SSM approach. Across quarterly analyses, the difference in IC estimates did not evolve significantly between 2017Q2, when the first two events were reported, and the end of the analysis period.

**FIGURE 7:**
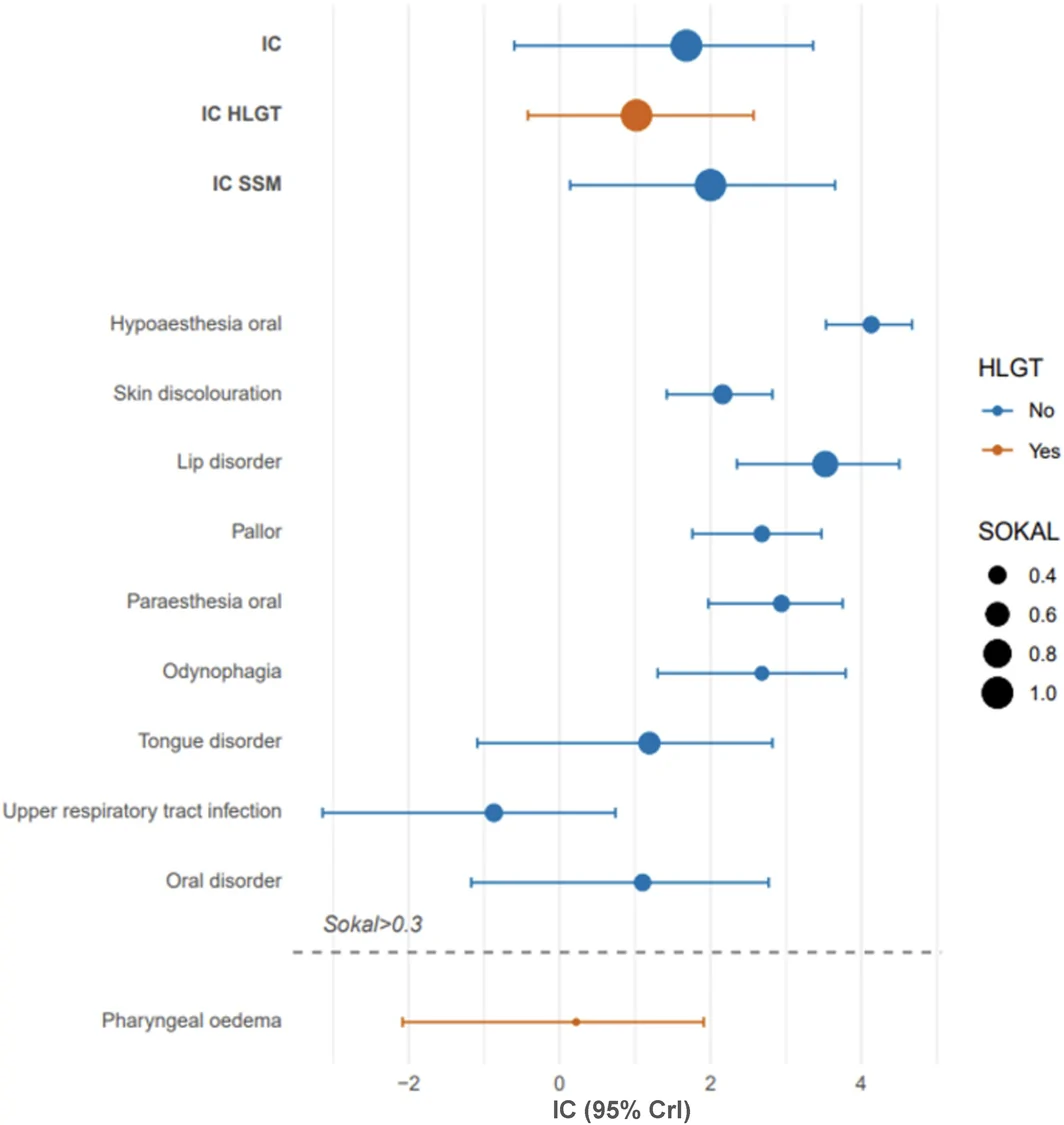
Graphical representation of PTs contributing to Bayesian borrowing in IC SSM and IC HLGT for Example 3: *Pharyngeal erythema* (2017Q2). Error bars represent the 95% CrIs. The bubble size of the point estimates is proportional to the Sokal similarity with the PT of interest.

None of the PTs with a Sokal similarity above 0.3 were included in the same HLGT as the PT of interest, *Pharyngeal erythema*. Consequently, the Bayesian borrowing structure differed entirely between IC HLGT and IC SSM. Within the HLGT *Upper respiratory tract disorders (excluding infections)*, only one other PT, *Pharyngeal oedema* (Sokal similarity = 0.26), was represented in the data, with just two cases reported. The resulting IC estimate for IC HLGT was lower than that of the traditional IC for the PT of interest and accompanied by a narrower CrI.

In contrast, the IC SSM method incorporated a distinct set of PTs for Bayesian borrowing. The most influential PT in the IC SSM analysis was *Hypoaesthesia oral*, which, despite its modest Sokal similarity of 0.37 with *Pharyngeal erythema* contributed substantially to the MAP prior due to a higher count of 24 reported cases. Its IC point estimate was markedly elevated compared to that of *Pharyngeal erythema* (traditional IC). Similarly, the point estimates of the other five most influential PTs were also elevated, suggesting potential data conflict with *Pharyngeal erythema*. Consequently, the dynamic borrowing implemented in IC SSM provided limited Bayesian shrinkage. However this shrinkage was still sufficient to cause the lower limit of the corresponding CrI to slightly exceed zero, thereby incorrectly alerting for a potential safety concern.

Clinically, *Pharyngeal erythema* is more akin to a sign or symptom or is etiologically related to other PTs rather than representing a distinct diagnosis. The PTs included in the MAP prior of IC SSM (e.g., *Hypoaesthesia oral, Skin discolouration, Lip disorder, Pallor, Paraesthesia oral, Odynophagia, Tongue disorder, Upper respiratory tract infection*, and *Oral disorder*) are medically proximate, especially with several pertaining to oral‐related events. However, most of these PTs demonstrated limited Sokal similarity with *Pharyngeal erythema*. Among the six PTs with more than two reported cases, only *Lip disorder* exhibited relatively high Sokal similarity (0.69). This example underscores a potential limitation of the IC SSM method in contexts characterized by sparse data and low semantic similarity, where contributions from less similar PTs can disproportionately influence the signal.

#### 3.4.4 Example 4: Proctitis Ulcerative

The results for this illustrative example are presented in Figure 8. The forest plot displays only the 25 PTs with the highest influence for each of the IC SSM and IC HLGT analyses, although many additional PTs from other HLGTs—and with Sokal similarity above 0.3—were incorporated into the dynamic borrowing in the IC SSM model. In this case, both the IC HLGT and IC SSM methods generated an alert, thereby producing a false positive for *Proctitis ulcerative*, whereas the traditional IC method did not trigger an alert.

**FIGURE 8:**
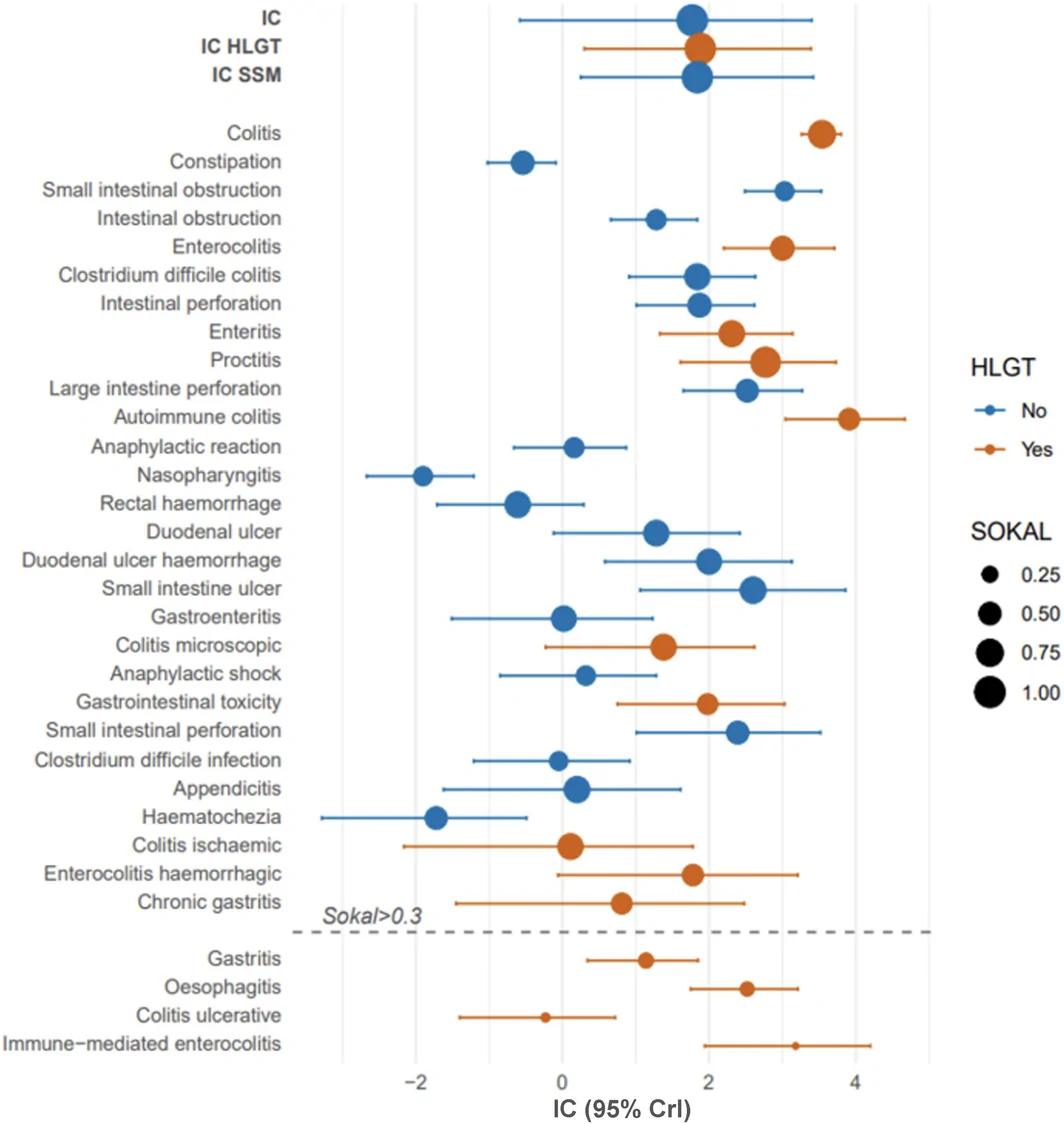
Graphical representation of PTs contributing to Bayesian borrowing in IC SSM and IC HLGT for Example 4: *Proctitis ulcerative* (2016Q1). Error bars represent the 95% CrIs. The bubble size of the point estimates is proportional to the Sokal similarity with the PT of interest.

The traditional IC analysis yielded an IC point estimate of 1.77 with a lower limit of −0.58, which falls below the alerting threshold. In contrast, the IC HLGT method, which borrows information from other PTs within the same HLGT *(Gastrointestinal inflammatory conditions)*, produced an IC point estimate of 1.88 with a lower limit of 0.30, thereby triggering an alert. Similarly, the IC SSM method reported an IC point estimate of 1.84 with a lower limit of 0.25, also prompting an alert.

A closer examination of the data reveals that several PTs included in the MAP prior for the IC SSM analysis were themselves positive controls, potentially biasing the results toward a false positive. For example, the PT *Colitis*—a positive control with 107 cases, an IC of 3.54, and a Sokal similarity of 0.77—contributed most strongly to the MAP prior. Additionally, other positive control PTs, such as *Intestinal obstruction* (24 cases, IC = 1.28, Sokal similarity = 0.39), and *Enterocolitis* (14 cases, IC = 3.00, Sokal similarity = 0.57), also contributed to the borrowing in the IC SSM model.

Certain PTs, such as *Proctitis*, exhibited very high Sokal similarity with the PT of interest (Sokal = 0.94). This high similarity increased the weight assigned to the MAP prior in the robust mixture prior of IC SSM, amplifying the borrowing effect. However, the individual point estimates of the many PTs included in the IC SSM borrowing exhibited considerable heterogeneity, increasing the variance of the MAP prior and reducing the degree of Bayesian shrinkage. Many of these PTs were clinically related signs and symptoms or AEs in close proximity rather than representing similar diagnoses, and they varied widely in their specificity relative to *Proctitis ulcerative*. By comparison, the IC HLGT model included only 14 PTs from the same HLGT, and although a similar (albeit slightly less pronounced) pattern was observed, the degree of Bayesian shrinkage was very similar between the two methods.

## 4 Discussion

This study introduced and evaluated a BDB method that integrates SSMs into DPA for SRSs of AEs. Using a robust MAP prior within a Bayesian hierarchical model, the proposed approach enables borrowing from MedDRA PTs based on their semantic similarity to the PT of interest. PTs with SSM values greater than 0.3 were included in the MAP prior, weighted by their SSM value divided by the estimated IC variance. A robustifying prior (Normal with mean = 0, SD = 2) was applied, and the maximum SSM was used to define the weight assigned to the MAP prior. Although, we limited our analysis to PTs with SSM values greater than 0.3 due to computational constraints, in principle, all PTs could be considered. Performance was compared to that of traditional IC analysis and IC analysis with BDB at the MedDRA HLGT level, using FAERS data. Evaluation relied on an automated, time‐stamped reference set derived from FDA safety labeling updates.

Our primary analyses, comparing methods at the conventional 2.5% alerting quantile (Figure 2), established a complex but promising performance profile for the IC SSM approach. It achieved the highest sensitivity (57.0%), outperforming both traditional IC (50.1%) and the simpler hierarchical borrowing method, IC HLGT (55.7%). Although, these differences were incremental, IC SSM clearly outperformed HLGT‐based borrowing in all performance analyses. Detailed case examples supported these observations by showing that SSM‐based borrowing provided more stable and contextually relevant estimates than HLGT‐based approaches. However, the primary configuration of IC SSM exhibited lower precision (14.5%) than traditional IC (16.1%), resulting in a slightly lower overall F1‐score and Youden's Index. The most striking finding from the primary analysis was the substantial timeliness advantage: IC SSM detected TPs, on average, 1.75 quarters earlier than traditional IC. This observation, coupled with the patterns seen in the quarterly analyses, prompted a series of post hoc investigations to determine if IC SSM's benefits were more fundamental than a simple shift along the precision‐recall curve.

These post hoc analyses explored the sensitivity‐precision trade‐off from several angles. When strategies to enhance precision were explored, IC SSM demonstrated a more favorable performance profile. For instance, when raising the alerting threshold of the 95% CrI—a practical step when signal management resources are constrained—IC SSM's performance degraded less sharply than that of traditional IC, surpassing it on Youden's index at a threshold of 0.2 (Supporting Information Figure S2). Furthermore, tuning the alerting quantile showed that a stricter IC SSM configuration (IC SSM q0.01) could achieve both higher sensitivity and higher precision than the standard traditional IC. Conversely, a precision‐matched comparison revealed that a relaxed traditional IC (IC q0.05) still failed to match the sensitivity of the primary IC SSM configuration (55.6% vs. 57.0%), suggesting the performance of IC SSM is not readily replicated through parameter tuning. This highlights a key limitation of the traditional method, as attaining comparable recall by other means would require lowering its alert threshold to counterintuitive levels, even potentially below zero, where an IC value would misleadingly suggest a protective effect.

Further post hoc investigation into the source of the timeliness advantage indicated that IC SSM's superiority is most pronounced in the sparse‐data settings characteristic of early surveillance. The analysis stratified by case counts (Table 3) showed that for product‐event pairs with three or fewer cases, IC SSM achieved a substantially higher F1‐score than traditional IC (0.141 vs. 0.098), driven by a large gain in sensitivity (0.260 vs. 0.109) and an improvement in precision (0.096 vs. 0.089). A final exploratory analysis of the timeliness data confirmed this, revealing that a significant proportion of the signals detected earlier by IC SSM occurred in these low‐count strata.

Taken together, these primary and post hoc findings suggest that IC SSM's higher sensitivity is not simply the result of a trade‐off. Instead, by enabling the robust integration of information from semantically related terms the method provides a more favorable performance profile. This is especially true in the critical early surveillance window, where it can be both more sensitive and more precise than the standard method. This indicates that IC SSM offers a unique advantage that is not attainable by simple parameter adjustments to the traditional IC method.

It is important to recognize that the consequences of false negatives and false positives are not equivalent, especially given the serious implications of missing a true safety signal. Although, alternative composite metrics that differentially weight recall and precision are available, we chose to focus on conventional performance measures for clarity and transparency. Using standard metrics provides a conservative assessment of IC SSM's performance; any weighting that prioritizes recall would further emphasize its comparative advantages over traditional IC.

The benefits of IC SSM were clearest in cases where the PT of interest belonged to broad or clinically diffuse HLGTs such as *Infections—pathogen unspecified* or *General system disorders NEC*. In these scenarios, SSMs enabled selective borrowing from PTs outside the HLGT that were clinically relevant and statistically informative, leading to more accurate and stable estimations. For instance, in the analysis of *Mouth ulceration*, SSM‐based borrowing included PTs with IC values more aligned with the target PT than those included under HLGT grouping. These findings underscore how IC SSM adapts borrowing based on semantic proximity, supporting more accurate and stable estimation. The practical consequence of this mechanism is evident in the illustrative examples. For the PT *Meningitis*, signal detection occurred three quarters earlier than with traditional IC. For the PT *Mouth ulceration*, the signal was identified 2.5 years sooner. These quantifiable gains in timeliness underscore the method's practical value, especially given that early detection is a primary goal of PV. However, other examples also highlighted potential reasons for false positives. In one instance, the target PT was a clinical sign (*Pharyngeal erythema*) that is more akin to a symptom or an etiological indication rather than a distinct diagnosis. The MAP prior of the IC SSM incorporated PTs that were medically proximate—many pertaining to oral‐related events—though with limited similarity. However, some had a high number of reported cases and consequently carried significant weight in the borrowing process, accidentally generating an alert. In Example 4 (*Proctitis ulcerative*), the MAP prior incorporated a large number of PTs, many of which were clinically related signs, symptoms, or AEs that were in close anatomical or etiological proximity to the target PT but did not represent truly similar diagnoses. While most of the PTs in the MAP prior exhibited low similarity to the target PT, one PT (*Proctitis*) had a high Sokal similarity with the PT of interest (Sokal = 0.94). This high similarity increased the relative weight of the MAP prior within the mixture prior, amplifying its influence on the analysis. The borrowing PTs varied widely in their specificity relative to the target PT, *Proctitis ulcerative*, and displayed considerable heterogeneity in their IC estimates. This heterogeneity contributed to an increase in the variance of the MAP prior, which, in turn, reduced the degree of Bayesian shrinkage. Despite the limited shrinkage achieved relative to the large number of PTs included, the borrowing was still sufficient to trigger an alert.

Sensitivity analyses further clarified the effects of tuning key parameters: The lower limit of SSM (minSSM), the prior weight w, and the standard deviation of the robustifying prior σ. Varying the threshold of minSSM below 0.6 had minimal impact on IC SSM performance. Increasing w boosted sensitivity but decreased specificity and precision for both IC SSM and IC HLGT. While F1 scores declined at higher weights, the configuration using max(SSM)—our reference setting—produced the highest F1 score overall. Across all w values, IC SSM outperformed IC HLGT, though traditional IC retained the highest F1 score. Similarly, decreasing σ increased sensitivity but reduced specificity. IC SSM consistently outperformed IC HLGT in both F1 score and Youden's index across all σ values, and achieved higher Youden's index than traditional IC at σ<2, where the influence of similar PTs is maximized and posterior uncertainty is minimized.

These results are broadly consistent with previous findings. The IMI‐PROTECT study [22] reported a mean sensitivity of 0.30 for IC—substantially lower than our results for both traditional IC (0.50) and IC SSM (0.57). Their slightly higher precision (0.18, vs. 0.14 for IC SSM) resulted in a marginally lower F1 score (0.225 vs. 0.243 and 0.231 for our IC and IC SSM, respectively). These differences likely reflect the use of older data, other SRS sources, and a smaller, non‐time‐stamped reference set. Because their study did not evaluate the detection of unknown AEs, comparability is limited. Evaluation of vigiRank [59], which combines DPA with AE case‐level features, reported even higher performance (sensitivity > 60%, specificity > 70%, AUC > 65%). However, these results are not directly comparable due to differences in methodology, reference sets, and data sources, underscoring the value of evaluating methods within a single consistent setting.

A persistent challenge in PV is selecting the appropriate level of specificity for AE definitions, as definitions that are too broad or too narrow can obscure true safety signals [28, 30]. Tree‐based scan statistics address this by testing across all hierarchical levels of a terminology with multiplicity adjustment [60], but they remain constrained by discrete, fixed taxonomies and have not demonstrated consistent advantages over traditional DPAs [61]. In contrast, our BDB approach with IC SSM departs fundamentally from this paradigm by weighting evidence according to semantic similarity rather than hierarchical adjacency. This continuous, data‐driven borrowing of strength across clinically related terms allows IC SSM to capture meaningful relationships that rigid tree‐based methods inherently overlook.

There are important limitations to our work. While our model draws inspiration from meta‐analytic re‐weighting strategies based on quality scores—some of which have been criticized for producing biased effect estimates due to the non‐independence of quality scores from study features [62]—our use of ontology‐based SSMs ensures that MedDRA PTs are weighted independently of their potential reporting biases. Model parameters were selected empirically, and potential confounders such as age, sex, and report year were not adjusted due to inconsistent reporting and to the short period of analysis. Furthermore, the analyses exploring different alerting quantiles were conducted post hoc and should be considered exploratory. While valuable for investigating the method's performance characteristics, these specific parameter settings would require independent validation before implementation in practice. Semantic similarity captures clinical relatedness but may not fully reflect spontaneous reporting patterns, which are influenced by patient behavior, media coverage, and reporting incentives. For example, the SSM between PT *Pharyngeal erythema* and PT *Pharyngeal oedema* illustrates that even clinically similar conditions of the same anatomic region can appear semantically distant under certain metrics. Despite representing related inflammatory processes, their Sokal distances were 0.43 in MedDRA, 0.40 in SNOMED, and 0.26 in the integrated MedDRA + SNOMED ontology—where lower values indicate greater distance. The nearest Sokal neighbors for *Pharyngeal erythema* (*Lip disorder*, *Inflammation of wound*, *Lip neoplasm*), and for *Pharyngeal oedema* (*Angioedema*, *Swelling face*, *Face oedema*) capture partial phenotypic overlap but differ anatomically. This example underscores that ontology‐based semantic similarity may imperfectly model clinically intuitive relationships, particularly when hierarchical or lexical paths fail to encode fine‐grained anatomic or pathophysiologic associations. Finally, our analyses used all available data for parameter selection and model tuning. Although, no external validation set was used, we performed sensitivity analyses using resampled reference sets to evaluate robustness.

Despite these limitations, this study has several strengths. We employed a large, time‐stamped reference set derived from regulatory labeling changes [57, 63], enabling prospective evaluation of detection performance. The IC SSM framework builds on an established Bayesian method that is computationally efficient and scalable to large datasets. It also addresses a fundamental challenge in PV: Defining meaningful groupings of medical concepts. Unlike rule‐based groupings in MedDRA (e.g., SMQs or HLGTs), IC SSM uses continuous weights derived from semantic similarity that better reflect the nuanced relationships between clinical events. Although, alternative approaches for calculating distances between AE codes based on co‐reporting information, such as the vigiVec method [36] exist, we intentionally did not adopt these methods. An approach based exclusively on pre‐existing ontologies has the advantage of being independent of any specific data source used for safety surveillance or for computing semantic similarity, thereby facilitating its application across multiple data sources or in multimodal safety surveillance contexts, where harmonization is critical. This independence not only makes ontology‐based SSMs easier to apply across data sources, but also avoids the potential risk of bias from non‐random co‐reporting of AEs that can occur in AE reporting databases.

To our knowledge, this is the first implementation of SSM‐informed borrowing in DPA for AE signal detection in spontaneous reporting. As a hypothesis generation tool, it offers practical benefits for early detection of safety concerns. Future work could expand on this foundation by exploring alternative borrowing models, incorporating additional or transformed similarity metrics, or applying Bayesian inference directly to case‐level data. Novel SSMs could be developed to better reflect spontaneous reporting dynamics—for instance, by integrating co‐reporting patterns or biological pathways. Testing IC SSM with vigiVec‐derived similarities [36] or comparing it directly to vigiRank [59] may yield further insight. Moreover, combining exposure‐based and outcome‐based BDB methods may enhance signal detection. Broader application to other real‐world data sources—such as claims or electronic health records data—will help assess generalizability across diverse PV settings.

## 5 Conclusion

This study shows that semantic similarity‐informed borrowing can improve post‐marketing safety surveillance compared to the current standard approach of DPA. Compared to both the traditional IC approach—routinely used in PV—and borrowing at the MedDRA HLGT level, the IC SSM method has the ability to detect true positive signals earlier, with improved sensitivity and favorable trade‐offs in F1 score and Youden's index.

While SRSs such as FAERS are known to contain incomplete or inconsistent data, particularly in how events are coded and reported, our results suggest that leveraging semantic similarity between MedDRA terms offers a more robust and clinically meaningful basis for information sharing than rigid HLGT‐based inclusion criteria. Rather than relying on binary membership in predefined groupings, SSM allows for continuous, graded borrowing that reflects the spectrum of clinical similarity between events.

To our knowledge, this is the first implementation of SSM‐informed borrowing in DPA for AE signal detection in spontaneous reporting databases. Future research should aim to replicate these results in other datasets and regulatory contexts, and explore alternative Bayesian borrowing strategies, novel similarity measures, and integration with case‐level or real‐world data sources.

## Author Contributions

François Haguinet and Jeffery Painter contributed to the conceptual design of the study, authored the manuscript, and participated in its critical revision. Gregory Powell provided clinical expertise and reviewed and edited the manuscript. Andrea Callegaro contributed to the statistical methodology and reviewed and edited the manuscript. Andrew Bate brought forward the idea of the new approach, contributed to the conceptual design, and reviewed and edited the manuscript.

## Funding

GSK covered all costs associated with the conduct of the study and the development of the manuscript and the decision to publish the manuscript.

## Conflicts of Interest

This manuscript has not been submitted to, nor is under review at, another journal or other publishing venue. The authors have no competing interests to declare that are relevant to the content of this article. J.P., F.H., G.P, A.C., and A.B. are employed by GSK and hold financial equities.

## Supporting information




**Figure S1:** Quarterly evolution of the numbers of alerts and true positives (TPs). IC = Traditional IC, IC SSM q0.01 = IC SSM alerting with quantile of 1% corresponding to the lower limit of the 98% CrI >0, IC q0.05 = Traditional IC alerting with quantile of 5%. When not mentioned, the standard 2.5% quantile was used.
**Figure S2:** Performances for varying thresholds of the lower limit of the 95% CrI of IC. IC = Traditional IC. **Figure S3:** Performance analyses over the entire study period. IC = Traditional IC, IC SSM q0.01, IC SSM with an alerting quantile of 1% corresponding to the lower limit of the 98% CrI exceeding 0, IC SSM q0.025 = IC SSM alerting with quantile of 2.5%, IC q0.05 = Traditional IC alerting with quantile of 5%, IC q0.025 = Traditional IC alerting with quantile of 2.5%.
**Figure S4:** Distribution of the difference in time‐to‐detection of TPs between IC SSM with alerting quantile of posterior IC distribution of 1% and Traditional IC. GT5 = product‐event pairs with more than five cases at first alert, LE5 = product‐event pairs with five cases or less at first alert.
**Figure S5:** Distribution of the difference in time‐to‐detection of TPs between IC SSM and Traditional IC with alerting quantile of 5%. GT5 = product‐event pairs with more than five cases at first alert, LE5 = product‐event pairs with five cases or less at first alert.
**Figure S6:** IC SSM performances for varying thresholds of minimum SSM for inclusion in MAP prior. IC = Traditional IC.
**Figure S7:** IC SSM and IC HLGT performances for varying weight of the MAP prior in the robust MAP prior. SSM = IC SSM, IC = Traditional IC, and HLGT = IC HLGT.
**Figure S8:** Performances for varying σ=s in the vague prior. SSM = IC SSM, IC = Traditional IC, and HLGT = IC HLGT.

## Data Availability

Our aim is to make all of our source code publicly available. We have created the associated GitHub repositories in the meantime as place holders where applicable. Final code updates will be published after acceptance of the manuscript. The reference data used to conduct this study can be reproduced from the PVLens data processing pipeline available from: https://github.com/GSK‐Global‐Safety/pvlens. The semantic similarity server and supporting libraries are published here: https://github.com/GSK‐Global‐Safety/semantic‐similarity. The final IC‐SSM processing pipeline code will be released here: https://github.com/GSK‐Global‐Safety/icssm.

## ENDNOTES

^1^
It should be noted that sales and prescription data are routinely used in other contexts, such as to establish denominators in PSURs and PBRERs; the distinction here is that they are less commonly used directly in disproportionality‐based signal detection.

^2^

https://github.com/bips‐hb/pvm/blob/master/R/BCPNN.R.

^3^
In each example, the products are distinct from one another.
