# Supplementary material for: Semantic Similarity‐Informed Bayesian Borrowing for Quantitative Signal Detection of Adverse Events
Source: Stat Med. 2026 Oct 15;45(23-24):e70757. doi: 10.1002/sim.70757 (PMC13673117; doi:10.1002/sim.70757)
Supplement: Supplementary file 1 [file SIM-45-0-s001.pdf]

APPENDIX

800

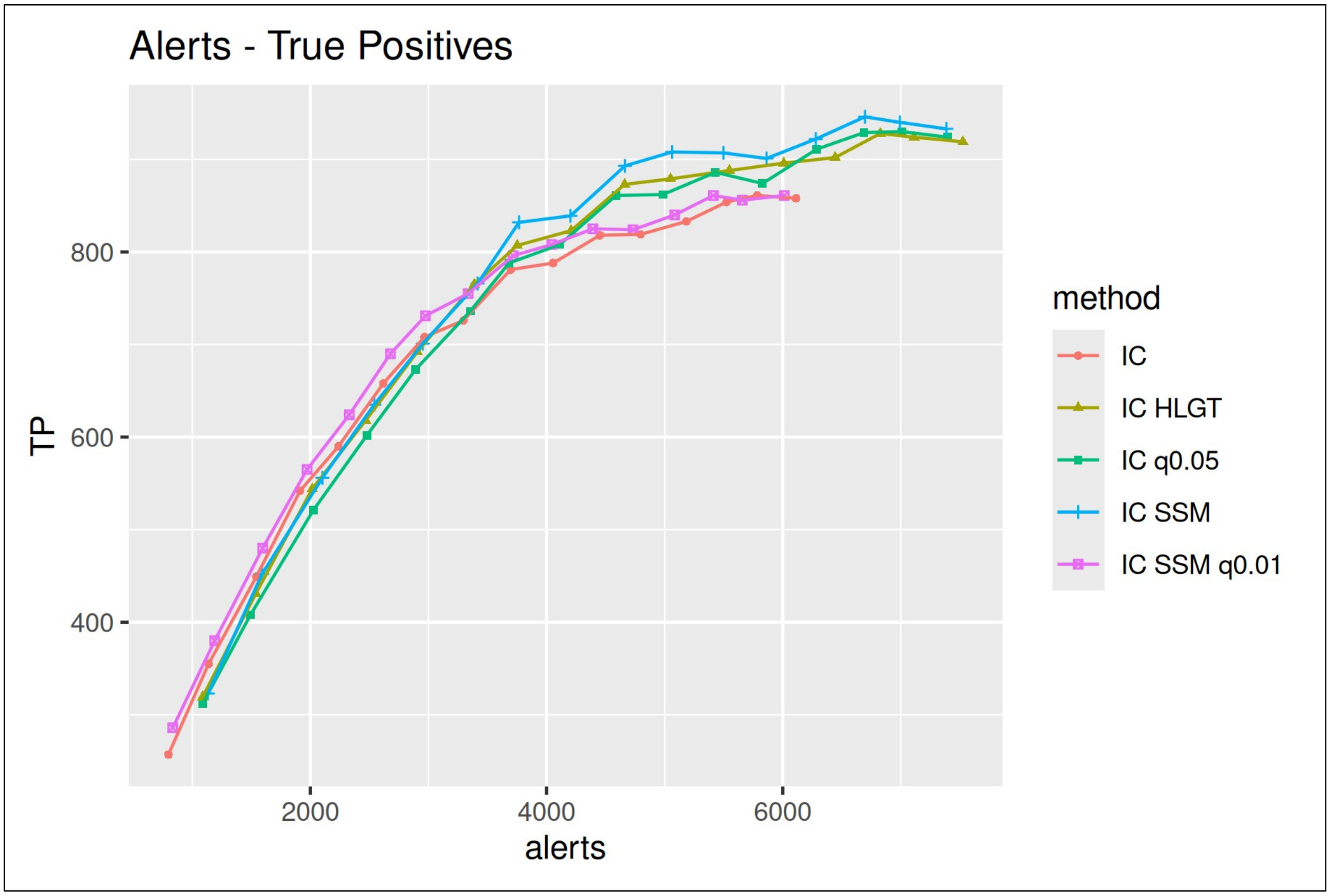

FIGURE S1
Quarterly evolution of the numbers of alerts and true positives (TPs). IC = Traditional IC, IC SSM $q0.01$ = IC SSM alerting with quantile of 1% corresponding to the lower limit of the 98% CrI > 0, IC q0.05 = Traditional IC alerting with quantile of 5%. When not mentioned, the standard 2.5% quantile was used.

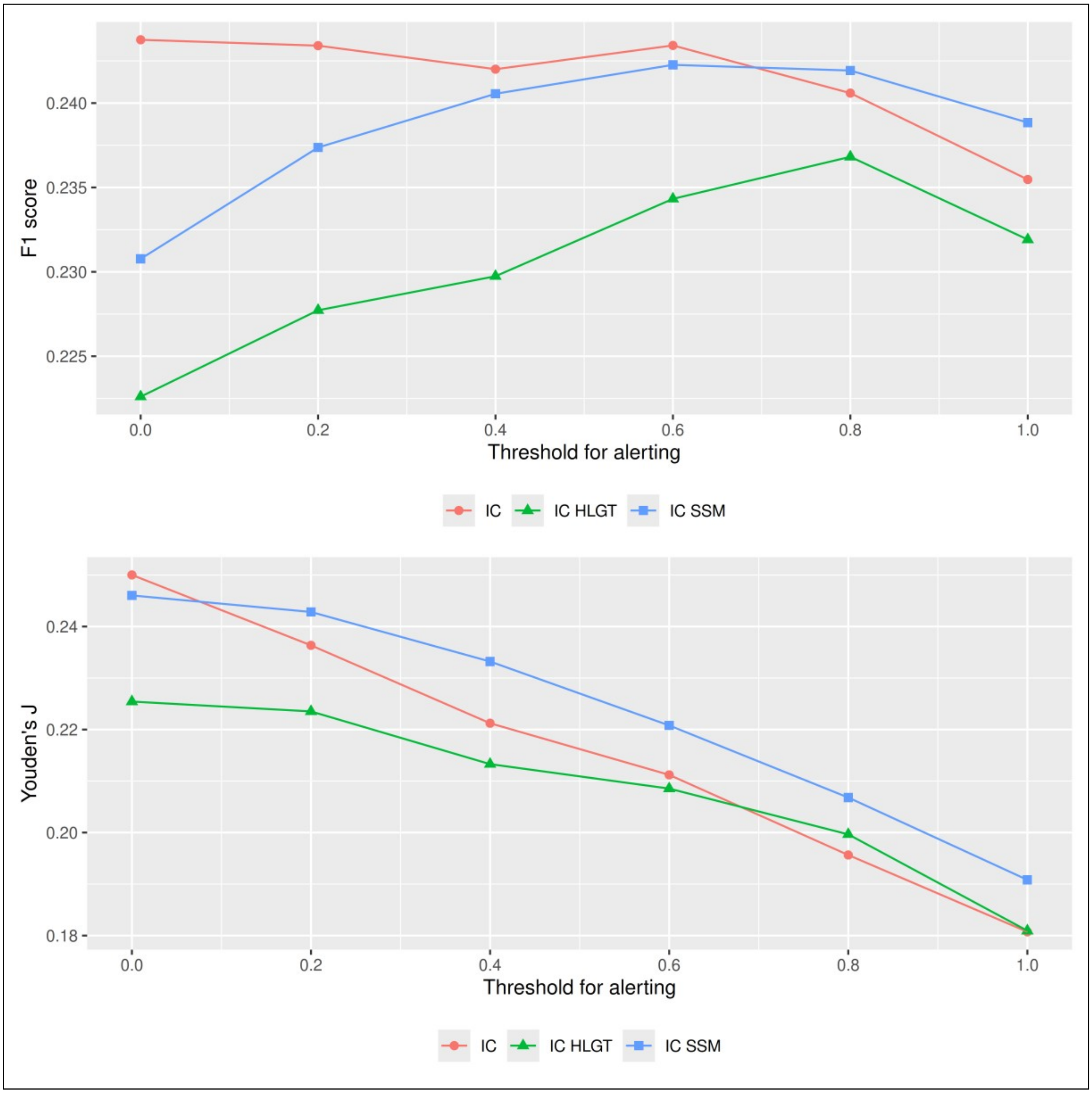

FIGURE S2 Performances for varying thresholds of the lower limit of the 95% CrI of IC. IC = Traditional IC.

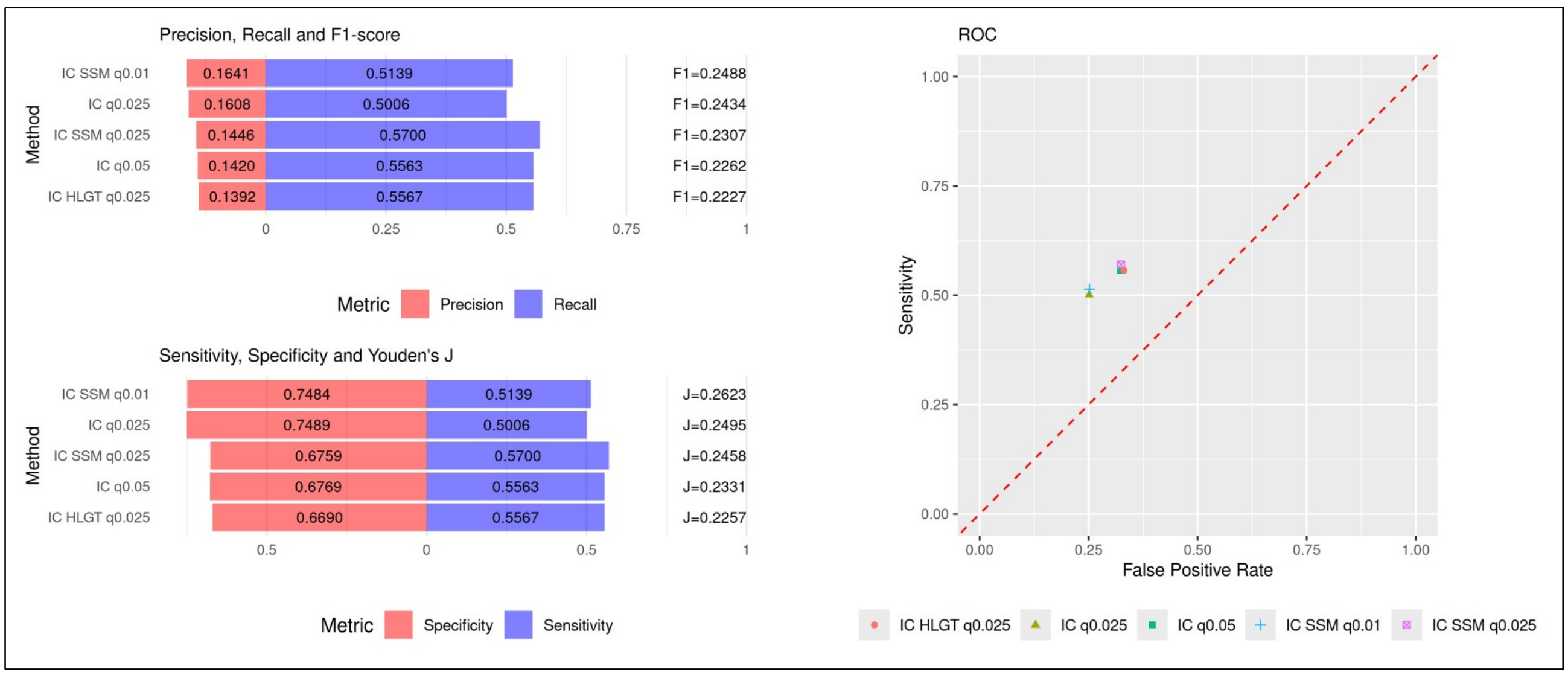

FIGURE S3

Performance analyses over the entire study period. IC = Traditional IC, IC SSM $q0.01$, IC SSM with an alerting quantile of 1% corresponding to the lower limit of the 98% CrI exceeding 0, IC SSM $q0.025$ = IC SSM alerting with quantile of 2.5%, IC $q0.05$ = Traditional IC alerting with quantile of 5%, IC $q0.025$ = Traditional IC alerting with quantile of 2.5%.

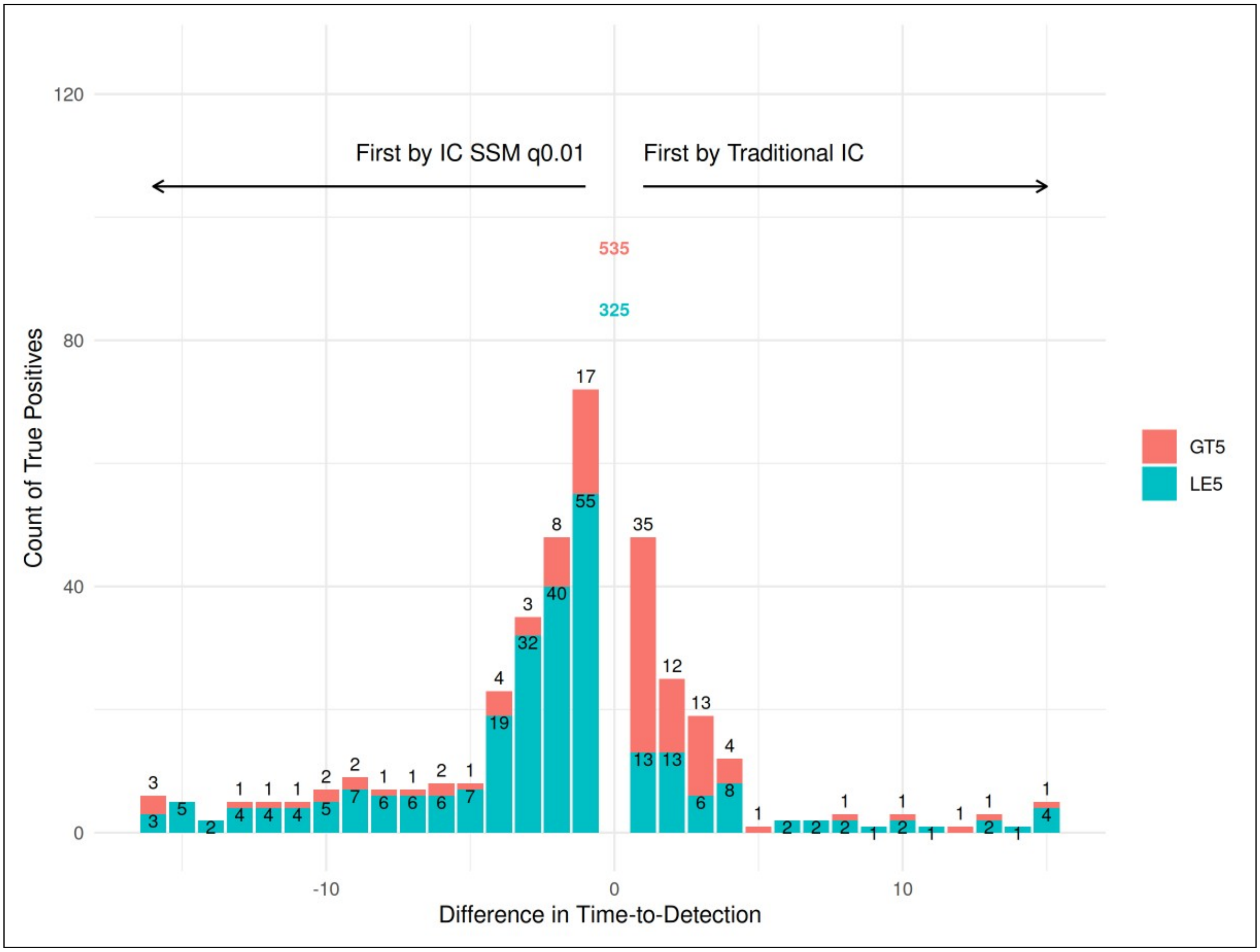

FIGURE S4
Distribution of the difference in time-to-detection of TPs between IC SSM with alerting quantile of posterior IC distribution of 1% and Traditional IC. GT5 = product-event pairs with more than five cases at first alert, LE5 = product-event pairs with five cases or less at first alert.

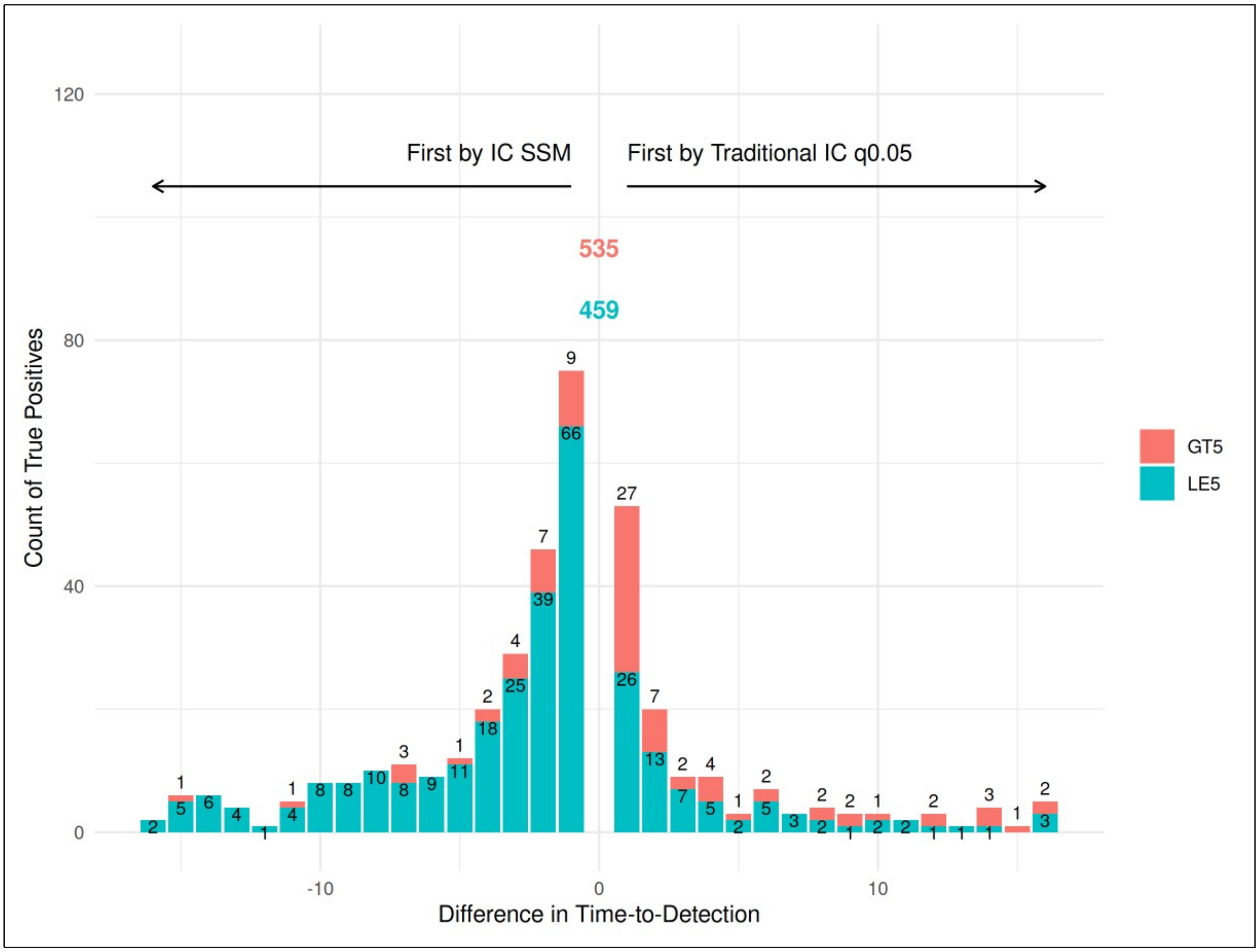

FIGURE S5
Distribution of the difference in time-to-detection of TPs between IC SSM and Traditional IC with alerting quantile of 5%. GT5 = product-event pairs with more than five cases at first alert, LE5 = product-event pairs with five cases or less at first alert.

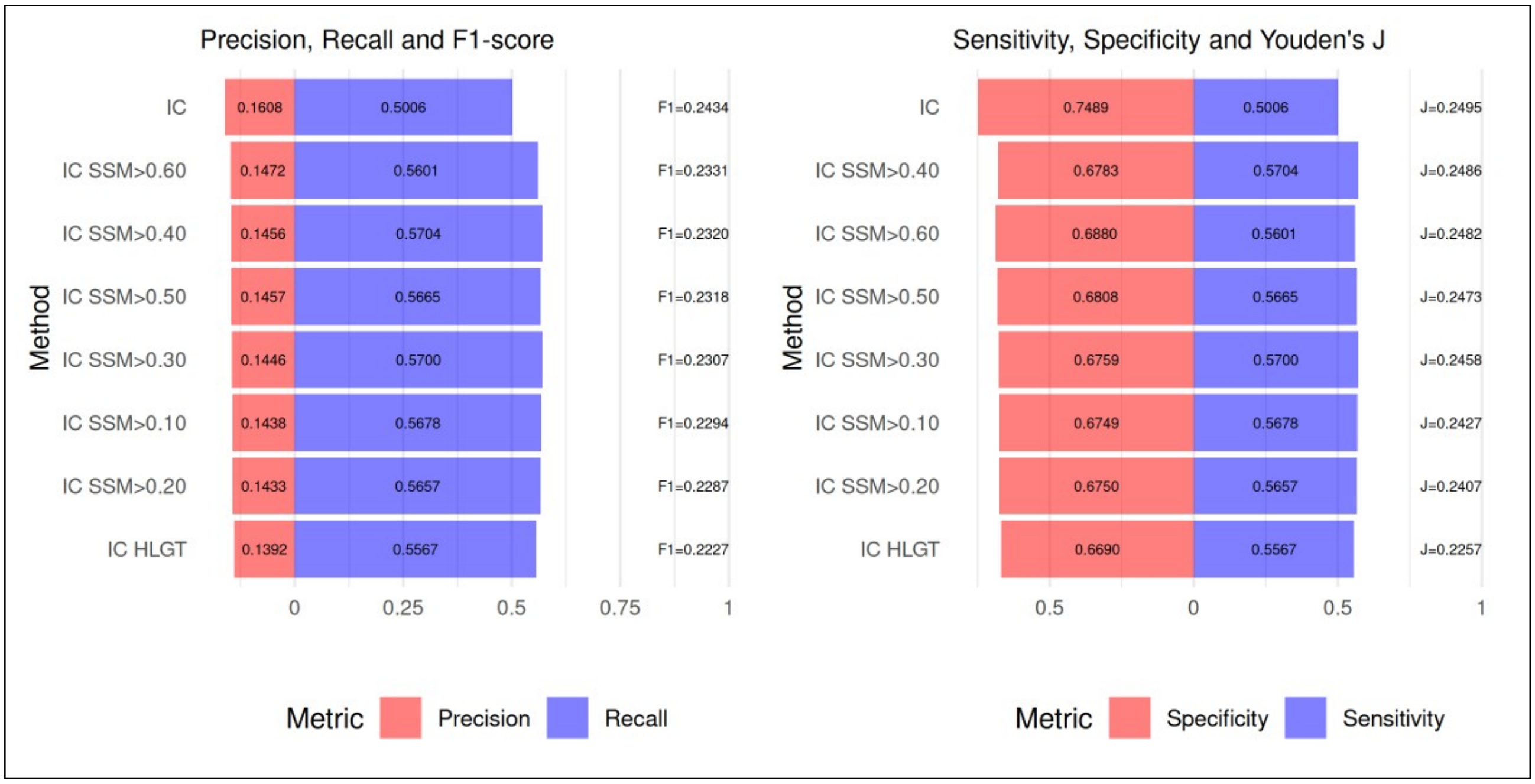

FIGURE S6

IC SSM performances for varying thresholds of minimum SSM for inclusion in MAP prior. IC = Traditional IC.

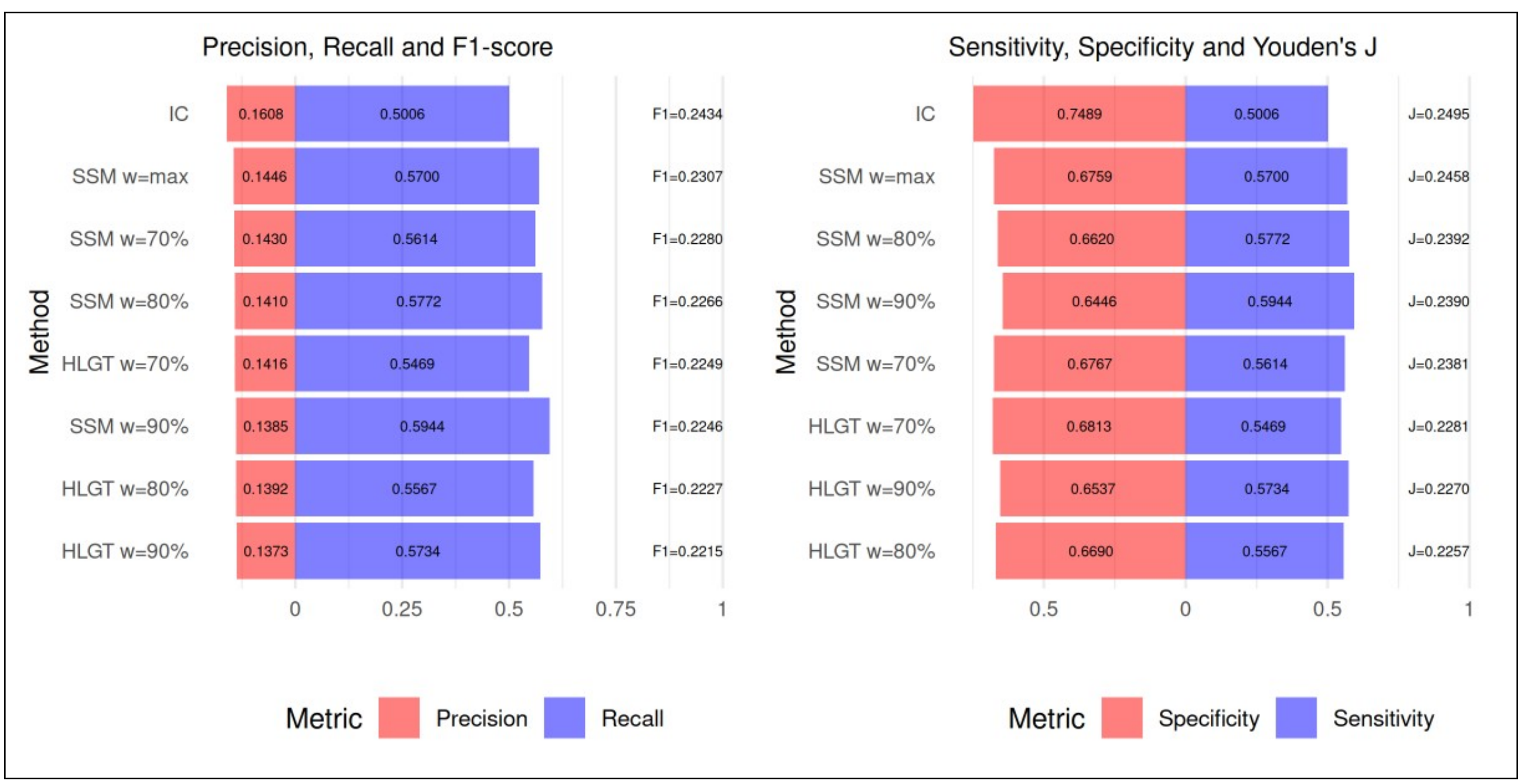

FIGURE S7

IC SSM and IC HLGT performances for varying weight of the MAP prior in the robust MAP prior. SSM = IC SSM, IC = Traditional IC, and HLGT = IC HLGT.

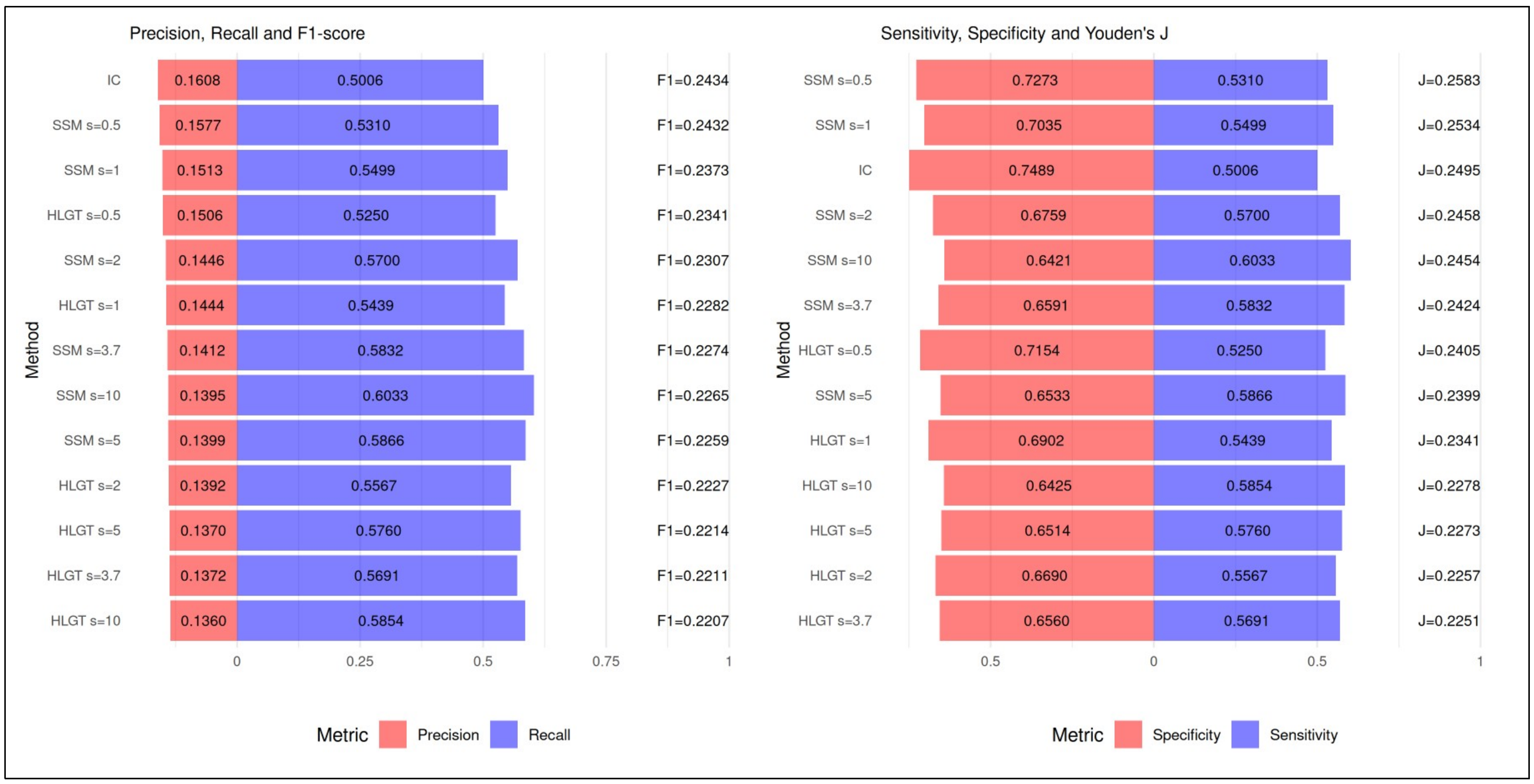

FIGURE S8

Performances for varying $\sigma = s$ in the vague prior. SSM = IC SSM, IC = Traditional IC, and HLGT = IC HLGT.
